# Dynamic glycolytic reprogramming effects on dendritic cells in pancreatic ductal adenocarcinoma

**DOI:** 10.1186/s13046-024-03192-8

**Published:** 2024-09-30

**Authors:** Bo Zhang, Kenoki Ohuchida, Chikanori Tsutsumi, Yuki Shimada, Yuki Mochida, Koki Oyama, Chika Iwamoto, Nan Sheng, Shuang Fei, Koji Shindo, Naoki Ikenaga, Kohei Nakata, Yoshinao Oda, Masafumi Nakamura

**Affiliations:** 1 Department of Surgery and Oncology, Graduate School of Medical Sciences, Kyushu University, 3-1-1 Maidashi, Higashi-Ku, Fukuoka, 812-8582 Japan; 2 Department of Anatomic Pathology, Pathological Sciences, Graduate School of Medical Sciences, Kyushu University, Fukuoka, 812-8582 Japan

**Keywords:** Glycolysis, Pancreatic ductal adenocarcinoma, Tumor microenvironment, Dendritic cell, Antigen-presenting function

## Abstract

**Background:**

Pancreatic ductal adenocarcinoma tumors exhibit resistance to chemotherapy, targeted therapies, and even immunotherapy. Dendritic cells use glucose to support their effector functions and play a key role in anti-tumor immunity by promoting cytotoxic CD8^+^ T cell activity. However, the effects of glucose and lactate levels on dendritic cells in pancreatic ductal adenocarcinoma are unclear. In this study, we aimed to clarify how glucose and lactate can impact the dendritic cell antigen-presenting function and elucidate the relevant mechanisms.

**Methods:**

Glycolytic activity and immune cell infiltration in pancreatic ductal adenocarcinoma were evaluated using patient-derived organoids and resected specimens. Cell lines with increased or decreased glycolysis were established from KPC mice. Flow cytometry and single-cell RNA sequencing were used to evaluate the impacts on the tumor microenvironment. The effects of glucose and lactate on the bone marrow-derived dendritic cell antigen-presenting function were detected by flow cytometry.

**Results:**

The pancreatic ductal adenocarcinoma tumor microenvironment exhibited low glucose and high lactate concentrations from varying levels of glycolytic activity in cancer cells. In mouse transplantation models, tumors with increased glycolysis showed enhanced myeloid-derived suppressor cell infiltration and reduced dendritic cell and CD8^+^ T cell infiltration, whereas tumors with decreased glycolysis displayed the opposite trends. In three-dimensional co-culture, increased glycolysis in cancer cells suppressed the antigen-presenting function of bone marrow-derived dendritic cells. In addition, low-glucose and high-lactate media inhibited the antigen-presenting and mitochondrial functions of bone marrow-derived dendritic cells.

**Conclusions:**

Our study demonstrates the impact of dynamic glycolytic reprogramming on the composition of immune cells in the tumor microenvironment of pancreatic ductal adenocarcinoma, especially on the antigen-presenting function of dendritic cells.

**Supplementary Information:**

The online version contains supplementary material available at 10.1186/s13046-024-03192-8.

## Introduction

Pancreatic cancer is one of the most lethal solid malignancies, with over 95% of cases being pancreatic ductal adenocarcinoma (PDAC). It is well-known that PDAC exhibits resistance to various forms of chemotherapy, targeted therapy, and even immunotherapy. Moreover, only approximately 15%–20% of patients are eligible for surgery at the time of diagnosis, which contributes to it being one of the deadliest cancers, with a 5-year survival rate of approximately 12% [1, 2]. The PDAC tumor microenvironment (TME) is a major contributor to its lethality. It is characterized by an extensive expansion of stromal fibroblasts and the associated extracellular matrix deposition, leading to increased interstitial fluid pressure and collapse of small arteries and capillaries. These effects result in poor vascular perfusion and reduced availability of nutrients in the TME. Cancer cells therefore exploit what is known as “metabolic reprogramming” to meet their energy demands and promote malignant behavior [3, 4]. Notably, oncogenic KRAS mutations are present in approximately 95% of PDAC tumors and are required for PDAC development. KRAS mutations promote the optimal conditions for cancer cell growth by elevating the uptake of glucose and glycolytic intermediates, fatty acids, and glutamine [5], commonly manifesting as enhanced aerobic glycolysis, known as the Warburg effect [6]. This effect is a prominent feature of many cancers and is characterized by active glycolysis and increased lactate production, even with an adequate oxygen supply.

Dendritic cells (DCs) use glucose to support their effector functions, with conventional dendritic cells (cDC) consisting of cDC1 and cDC2 subsets. These two subsets play crucial roles in anti-cancer immunity by promoting the activation of cytotoxic CD8^+^ T cells and CD4^+^ T cells, respectively [7, 8]. Preclinical mouse model studies have shown that cDC1 cells are necessary for T cell-mediated tumor regression and a therapeutic response to immune checkpoint blockade (ICB). In many human cancers, the number of cDC1 cells significantly impacts patient survival rates and clinical responses to ICB [9, 10]. Metabolic competition and glucose restriction, as metabolism-related mechanisms of immunosuppression, have been shown to cause major glycolytic and bioenergetic defects in T cells, resulting in decreased secretion of interferon gamma (IFN-γ) [11–13]. However, the impacts of nutrient restriction on the functions of pattern recognition receptors and other receptor systems in DC subsets remain to be explored [7, 8]. Relative to other tumor types, PDAC tumors have a low number of DCs in the TME with a poor antigen-presenting capacity. Currently, research on the DC antigen-presenting function mainly focuses on the interaction of immune cells and the influence of cytokines [14, 15]. The impact of metabolic reprogramming in tumors on this antigen-presenting function is still unclear.

Changes in nutrient and metabolite levels in the TME can influence tumor-immune interactions, as well as immune cell function and subset differentiation [16, 17]. However, studies on the effects of glucose and the metabolic byproduct lactate on immune cells in the PDAC TME are limited. Here, we investigated the impacts of cancer cells with altered glycolysis on the composition and function of immune cells in the TME. We orthotopically transplanted mouse PDAC cells with increased and decreased glycolysis in mouse models, particularly focusing on the metabolic mechanisms affecting DC functions. Our results reveal that cancer cells with increased glycolysis can transform the TME into a low-glucose and high-lactate environment, leading to a reduced number of DCs and decreased antigen-presenting function. Thus, inhibiting cancer cell glycolysis may offer a potential strategy to improve anti-tumor immune responses in PDAC.

## Materials and methods

### Mouse PDAC cells

Primary cultures of mouse PDAC cell lines were established from primary tumors in KPC mice (*Pdx1-Cre*; *LSL-Kras*^ *G12D*^; *Trp53*^ *R172H/*+^) using the outgrowth method [18] and cultured in Dulbecco’s Modified Eagle Medium (DMEM; Sigma-Aldrich, D5523; St. Louis, MO, USA) supplemented with 10% fetal bovine serum (FBS) and 5.5 mM glucose. The cells were cultured at 37 °C in a humidified atmosphere containing 10% CO_2_. Increased glycolysis (IG) cancer cells were established from mouse PDAC cells cultured for weeks in DMEM supplemented with 1% FBS, which contained 50 mM glucose. The shLDHA cancer cells with decreased glycolysis were established from mouse PDAC cells transfected with *Ldha* short hairpin RNA (shRNA) lentivirus particles (shLDHA-1: 5′-GTACGTCCATGATGCATATCT-3′; shLDHA-2: 5′-GTTCCCAGTTAAGTCGTATAA-3′; shLDHA-3: 5′-CGTGAACATCTTCAAGTTCAT-3′; Sigma-Aldrich). The shNC (negative control) cancer cells were established from mouse PDAC cells transfected with MISSION® pLKO.1-puro non-mammalian shRNA control transduction particles (Sigma-Aldrich, SHC016V-1EA). The *Ldha* gene knockdown efficiency was confirmed by western blot analysis and quantitative reverse transcription polymerase chain reaction (qRT-PCR).

### Mice

Mice were on 12-h light–dark cycles and maintained at 20 °C–25 °C and 30%–70% humidity. C57BL/6 and BALB/c-nu female mice were purchased from CLEA Japan (Tokyo, Japan). For the orthotopic transplantation experiments, mouse PDAC cells (1 × 10^5^ Control, IG, shNC, or shLDHA cells) were suspended in 50 µL phosphate buffered saline (PBS), then orthotopically injected into the pancreas of 5-week-old C57BL/6 female mice or 5-week-old BALB/c-nu female mice. All mice were sacrificed 18 to 20 days after orthotopic injection.

### Bone marrow-derived dendritic cell (BM-DC) generation

BM-DCs were induced as described previously [19]. In brief, BM cells were flushed from 8–12-week-old C57BL/6 female mouse tibias and femurs, and red blood cells were lysed using Lysing Buffer (BD Biosciences, 555,899; San Jose, CA, USA). After being washed and centrifuged at 400 × g, BM cells were cultured in Roswell Park Memorial Institute (RPMI) 1640 media (Sigma-Aldrich, R8755) supplemented with 10% FBS, 11 mM glucose, 55 μM 2-mercaptoethanol (Thermo Fisher Scientific, 21,985,023; Waltham, MA, USA), 5 ng/mL murine recombinant granulocyte/macrophage-colony stimulating factor (GM-CSF) (Wako, 077–04674; Osaka, Japan), and 200 ng/mL murine recombinant Flt3-Ligand (FLT3-L) (PeproTech, 250-31L; Cranbury, NJ, USA) at 37 °C in a humidified atmosphere containing 5% CO_2_ for 9 days. For analysis of the BM-DC differentiation rate, BM cells were cultured in RPMI 1640 media supplemented with 10% FBS, 11 mM glucose, and 0 mM L-LA (Sodium L-lactate, Sigma-Aldrich, L7022-5G) or 10% FBS, 1 mM glucose, and 15 mM L-LA. Recombinant cytokines were added as described above. The differentiation rate was analyzed by flow cytometry (FCM) after 9 days of culturing.

### Air–Liquid Interface Three-Dimensional (ALI-3D) co-culture of mouse PDAC cells and BM-DCs

The ALI-3D co-culture model was established by combining methods from previous studies [20, 21]. In brief, mouse PDAC cells dissociated into single cells and collected induced BM-DCs were mixed at a ratio of 1:5 (1 × 10^5^ mouse PDAC cells: 5 × 10^5^ BM-DCs). Mixed cells (5 × 10^5^ mouse PDAC cells and 2.5 × 10^6^ BM-DCs) were suspended in 500 μL RPMI 1640 media supplemented with 10% Matrigel (Corning, 356,231; Corning, NY, USA) and 10% FBS. Next, 100 μL of the cell suspension was seeded into the round-bottom ultra-low attachment 96-well plate. The plate was centrifuged at 400 × g for 3 min to aggregate the cells, then incubated overnight at 37 °C in a humidified atmosphere containing 5% CO_2_. The next day, after preparing the culture dishes and collagen gel, the inner dish (Merck Millipore, PICM03050; Darmstadt, Germany) was inserted into the outer dish (Thermo Fisher Scientific, 150,462). Then, collagen gel matrices of cellmatrix type I-A (Wako, 637–00653), concentrated culture solution RPMI-1640 culture solution (Wako, 633–29,651), and reconstitution buffer (Wako, 635–00791) were mixed at a ratio of 8:1:1 on ice. Next, 1 mL of collagen gel matrices were transferred to the inner dish and placed in a 37 °C incubator for 30 min to solidify. The aggregated cells were transferred onto the solidified collagen gel matrices using a 1000 μL pipette tip, then 1 mL of collagen gel matrices was transferred onto the aggregated cells and solidified in a 37 °C incubator for 15 min. Finally, the cells were cultured in 2 mL of RPMI 1640 media for one week. For single cell preparation, collagen gel matrices were dissociated with 200 units/mL collagenase IV (Worthington Biochemical, CLSS4; Lakewood, NJ, USA) at 37 °C for 30 min and trypsin was added by pipetting to dissociate the aggregated cells into single cells.

### Human PDAC tissue samples

Human PDAC tissue samples and normal pancreatic tissue samples (non-tumor areas from patients) were obtained from the Department of Surgery and Oncology, Kyushu University Hospital (Fukuoka, Japan). The patients for establishment of PDAC organoids provided written informed consent and the other patients for retrospective analysis waived written informed consent.

### Human PDAC organoids

All PDAC organoids (PDOs) were established from human PDAC tissue samples as described previously [22]. In brief, PDOs were cultured in a 24-well plate (Corning, 353,504) with growth factor-reduced Matrigel (Corning, 356,231) and niche media (see below) at 37 °C in a humidified atmosphere containing 10% CO_2_. The niche media contained 50% Dulbecco’s Modified Eagle Medium/Ham's F-12 (DMEM/F12) (Thermo Fisher Scientific, 12,634,010) and 50% Wnt3a, R-spondin1, Noggin-conditioned media (ATCC, L-WRN; Manassas, Virginia, USA) supplemented with 1 mM HEPES (Thermo Fisher Scientific, 15,630,080), 2 mM GlutaMAX (Wako, 35,050,061), 1 × Penicillin–Streptomycin (Thermo Fisher Scientific, 15,140,122), 1 × B27 (Thermo Fisher Scientific, 17,504,044), 10 mM Nicotinamide (Sigma-Aldrich, N0636), 1 mM N-acetyl-L-cysteine (Sigma-Aldrich, A9165), 50 ng/mL human recombinant EGF (PeproTech, AF-100–15), 0.5 mM A83-01 (R&D Systems, 2939; Minneapolis, MN, USA), and Y-27632 (Sigma-Aldrich, Y0503).

### Proliferation assay and ATP measurement

The CellTiter-Glo® Luminescent Cell Viability Assay (Promega, G7571; Madison, WI, USA) was used to evaluate the proliferation capacity and ATP levels of cells. For the proliferation assay, 1 × 10^3^ mouse PDAC cells/100 µL DMEM supplemented with 10% FBS and 5.5 mM glucose were seeded in a 96-well plate (Greiner Bio-One, 655,083). The luminescence was measured at the 0-h, 12-h, 24-h, 36-h, and 48-h timepoints. For measuring ATP levels, 2 × 10^4^ BM-DCs/100 µL RPMI 1640 media, supplemented with 10% FBS, 11 mM glucose, and 0 mM L-LA or 10% FBS, 1 mM glucose, and 15 mM L-LA, were seeded in a 96-well plate. The luminescence was measured using a microplate reader (TECAN, Infinite200; Männedorf, Switzerland) at the 0-h, 24-h, and 48-h timepoints.

### Glucose and lactate concentration measurements

The glucose and lactate concentrations in the culture supernatants and tumor tissues were quantified using the Glucose-Glo™ Assay (Promega, J6021) and Lactate-Glo™ Assay (Promega, J5021), respectively. To harvest the culture supernatant of 2D cells, 5 × 10^4^ cells/400 µL DMEM supplemented with 10% FBS and 5.5 mM glucose were seeded into a 24-well plate and cultured for 24 h. To harvest the culture supernatant of 3D cells, 1 × 10^5^ cells/50 µL Matrigel were seeded into a 24-well plate. After being solidified at 37 °C for 15 min, 400 µL of niche media was added and incubated for 72 h. Frozen tumor tissues were cut and normalized to a weight of 15 mg using the Premix 50 mM Tris (pH 7.5) buffer (Homogenization Buffer) with Inactivation Solution. Luminescence measurements were performed using a microplate reader (TECAN, Infinite200) following manufacturer instructions.

### Migration and invasion assays

For migration assays, 2 × 10^4^ mouse PDAC cells/250 µL DMEM supplemented with 2% FBS were seeded into 8 μm pores Transwell chambers (Corning, 353,097). Then, the chambers were inserted into a 24-well plate (Corning, 353,504) with 750 µL DMEM supplemented with 10% FBS. After being incubated for 24 h, the cells were fixed with 70% ethanol and stained with hematoxylin and eosin (H&E). For invasion assays, 8 μm pores Transwell chambers were coated with 20 μg Matrigel overnight and 5 × 10^4^ mouse PDAC cells/250 µL DMEM supplemented with 2% FBS were seeded into 8 μm pores Transwell chambers. Then, the chambers were inserted into a 24-well plate with 750 µL DMEM supplemented with 10% FBS. After being incubated for 48 h, the cells were fixed with 70% ethanol and stained with H&E. Images were acquired by BZ-X700 (KEYENCE; Osaka, Japan).

### Immunohistochemistry (IHC) and immunofluorescence (IF) staining

For IHC staining, formalin-fixed and paraffin-embedded tissues were cut at 4 μm thickness, then deparaffinized with xylene and ethanol. Endogenous peroxidase activity was blocked using methanol containing 0.3% hydrogen peroxidase at room temperature (RT) for 30 min. Antigen retrieval was performed in citrate buffer (pH 6.0) or Tris–EDTA buffer (pH 9.0) for 20 min in a pressure cooker. The slides were blocked with 3% BSA-PBS and incubated with primary antibodies at 4˚C overnight. The primary antibodies included: anti-CD8 human (150 µL, Nichirei Biosciences, 413,201; Tokyo, Japan), anti-CD11c human (1:500, Abcam, ab52632; Cambridge, UK), anti-LDHA human (1:400, Cell Signaling Technology, 3582 s; Danvers, MA, USA), anti-CD11b mouse (1:4000, Abcam, ab133357), anti-CD8a mouse (1:400, Cell Signaling Technology, 98941 s), anti-CD11c mouse (1:400, Cell Signaling Technology, 97,585), and anti-CD4 mouse (1:500, Abcam, ab183685), anti-αSMA mouse (1:100, Dako, M0851). The next day, after being washed, the slides were incubated with the EnVision System-HRP Labeled Polymer Anti-Rabbit (2 drops, Dako, K4003; Santa Clara, CA, USA) or Anti-Mouse (2 drops, Dako, Japan, K4001) secondary antibodies at RT for 60 min. Then, 3,3'-diaminobenzidine (Dako, K4065) was used for HRP detection. Sirius red staining was performed using the Picro-Sirius Red Stain Kit (SCY, PSR-1; Tokyo, Japan). Images were acquired by BZ-X700 (KEYENCE).

For IF staining, 1 × 10^6^ BM-DCs were cultured in RPMI 1640 media supplemented with 10% FBS, 11 mM glucose, and 0 mM L-LA or 10% FBS, 1 mM glucose, and 15 mM L-LA for 48 h. The cells were then suspended in a 1.5 mL tube with ice-cold PBS and fixed with 4% paraformaldehyde at 4˚C for 30 min. Then, the cells were washed with 0.1% BSA-PBS, blocked with 3% BSA-PBS at 4˚C for 30 min, and incubated with the anti-Tim23 (1:50, Santa Cruz Biotechnology, sc-514463; Dallas, TX, USA) primary antibody at 4˚C overnight. After being washed with 0.1% BSA-PBS, the cells were incubated with the Alexa Fluor™ 488 Goat anti-Mouse IgG (1:200, Thermo Fisher Scientific, A-11029) secondary antibody with 4′,6-Diamidino-2-phenylindole Dihydrochloride Solution (1:1000 DAPI, Wako, D523) at 4˚C for 60 min. After being washed, 1 × 10^4^ BM-DCs/100 µL PBS were centrifuged onto glass slides by Cytospin 4 (Thermo Fisher Scientific, A78300003). Images were acquired by BZ-X800 (KEYENCE). Five random fields were selected from each slide, excluding those with the highest and lowest cell counts. Positive cells for IHC staining and IF staining were examined and quantified by ImageJ software (National Institutes of Health, Bethesda, MD, USA). The LDHA staining intensity of tumor cells was graded using ImageJ as follows: 0^+^ (almost no staining), 1^+^ (weak), 2^+^ (moderate), or 3^+^ (strong). The assessment results were double-checked by the pathologist involved with this study.

### qRT-PCR

Total RNA was extracted from PDOs and mouse PDAC cells using the High Pure RNA Isolation kit (Roche, 11828665001; Basel, Switzerland). For two-step qRT-PCR of PDOs, total RNA samples were reverse transcribed into complementary DNA (cDNA) using the High-Capacity RNA-to-cDNA kit (Thermo Fisher Scientific, 4387406), then qRT-PCR was performed using the PowerUp™ SYBR™ Green Master Mix (Thermo Fisher Scientific, A25780) and CFX96 Touch Real-Time PCR Detection system (Bio-Rad, Hercules, CA, USA). One-step qRT-PCR of mouse PDAC cells was performed using the iTaq Universal SYBR Green One-Step Kit (Bio-Rad, 172–5150) and CFX96 Touch Real-Time PCR Detection system (Bio-Rad). Primers were purchased from Takara Bio (Kusatsu, Japan) and Sigma-Aldrich. The sequences of the primers are as follows: Human: *LDHA*, 5′-GGCAGATGAACTTGCTCTTGTTG-3′ (forward) and 5′-GACCAGCTTGGAGTTTGCAGTTA-3′ (reverse); *GLUT1* (SLC2A1), 5′-CATGACCATCGCGCTAGCA-3′ (forward) and 5′-AGAGTTCAGCCACGATGAACCA-3′ (reverse); *MCT4* (SLC16A3), 5′-CCAAGGCCGTCAGTGTCTTC-3′ (forward) and 5′-GTTCACGCACACACTGCAGAG-3′ (reverse); *β-ACTIN*, 5′- TGGCACCCAGCACAATGAA-3′ (forward) and 5′- CTAAGTCATAGTCCGCCTAGAAGCA-3′ (reverse). Mouse: *Ldha*, 5′-AGCTTCCATTTAAGGCCCCG-3′ (forward) and 5′-TCTTTTGAGACCGCTAGTGC-3′ (reverse); *Hk2*, 5′-TGATCGCCTGCTTATTCACGG-3′ (forward) and 5′-AACCGCCTAGAAATCTCCAGA-3′ (reverse); *mTOR*, 5′- ACCGGCACACATTTGAAGAAG-3′ (forward) and 5′- CTCGTTGAGGATCAGCAAGG-3′ (reverse); *β-actin*, 5′-CATCCGTAAAGACCTCTATGCCAAC-3′ (forward) and 5′- ATGGAGCCACCGATCCACA-3′ (reverse). The *β-ACTIN*/*β-actin* is a housekeeping gene used to normalize messenger RNA (mRNA) expression levels. The 2^−ΔΔCt^ calculation method was used to analyze data.

### Western blot analysis

Total protein was extracted from cells lysed in PRO-PREP Protein Extraction Solution (iNtRON Biotechnology, 17081; Seongnam-si, Gyeonggi-do, South Korea). Then, 30 μg of protein per sample was loaded onto Mini-PROTEAN TGX Precast Gels (Bio-Rad, 4561086). The Trans-Blot Turbo Transfer Starter System (Bio-Rad) was used to transfer proteins to Turbo Mini PVDF membranes (Bio-Rad, 1704156). After being blocked with Tris-buffered saline with 0.1% Tween® 20 detergent (TBST) buffer containing 5% milk, the membrane was incubated with anti-LDHA mouse (1:1000, Cell Signaling Technology, 2012S) and β-actin (1:2000, Cell Signaling Technology, 4970S) primary antibodies at 4 °C overnight. The next day, after being incubated with an anti-rabbit IgG secondary antibody (1:2000, Cell Signaling Technology, 7074) and washed with TBST buffer, Amersham™ ECL Select™ Western Blotting Detection Reagent (Cytiva, RPN2236; Tokyo, Japan) was added to the membranes and images were acquired using the ChemiDoc XRS System (Bio-Rad).

### FCM

Mouse PDAC tumors were sliced into small 2–4 mm pieces, then single cell suspensions were obtained using the Tumor Dissociation Kit (Miltenyi Biotec, 130–096-730; San Diego, CA, USA) at 37 °C for 40 min following the manufacturer’s instructions. After being filtered, the cells were incubated with the TruStain FcX™ (BioLegend, 101320; San Diego, CA, USA) blocking solution at 4 °C for 10 min. To assess the expression patterns of cell surface proteins, the samples were incubated with the following antibodies at 4 °C for 30 min: PE-Cy7 anti-CD4 (BD Biosciences, 563933), FITC anti-CD8 (BD Biosciences, 553031), PE anti-CD3 (BioLegend, 100206), APC/Cy7 anti-F4/80 (BioLegend, 123118), PerCP anti-CD45 (BD Biosciences, 557235), Brilliant Violet 421 anti-CD45 (BD Biosciences, 563890), FITC anti-CD45 (BioLegend, 103108), PE-Cy7 anti-CD11b (BD Biosciences, 561098), PE-anti-CD11c (BD Biosciences, 553802), FITC anti-H-2 (BioLegend, 125508), Alexa Fluor 647 anti-I-A/I-E (BioLegend, 107618), Alexa Fluor 647 anti-CD279 (BioLegend, 329910), Alexa Fluor 700 anti-CD86 (BioLegend, 105024), Alexa Fluor 700 anti-Ly-6G/Ly-6C (Gr-1) (BioLegend, 108422), PE-CF594 anti-CD80 (BD Biosciences, 562504), Brilliant Violet 711 anti-Ly-6C (BioLegend, 128037), and FITC anti-Ly-6G (BioLegend, 127605). After being washed, the cells were resuspended in 7-Aminoactinomycin D (7AAD) (BD Biosciences, 559925) in PBS for FCM analysis. The gate margins were determined by fluorescence minus one (FMO). Samples were acquired by FACSAria Fusion (BD Biosciences) and data were analyzed with FlowJo 10.5.3 software (BD Biosciences).

### 2-(N-(7-Nitrobenz-2-oxa-1,3-diazol-4-yl) Amino)-2-Deoxyglucose (2-NBDG) glucose uptake assay

Glucose uptake assays were performed by FCM. First, 1 × 10^5^ cells/well were seeded in a 6-well plate overnight. The cells were washed twice with PBS and subsequently cultured in glucose-free DMEM with 100 μM of 2-NBDG (Thermo Fisher Scientific, N13195) at 37 °C for 30 min. Then, the cells were maintained in ice-cold PBS to stop the reaction and protected from light until analysis. Unstained cells were used to determine the baseline glucose uptake levels. “Relative mean fluorescent intensity (MFI)” denotes the 2-NBDG MFI of stained samples relative to the matched unstained cells.

### Seahorse assay

The oxygen consumption rate (OCR) and extracellular acidification rate (ECAR) of mouse PDAC cells and BM-DCs were measured using a Seahorse assay (Agilent Technologies, Seahorse XFe24 analyzer; Santa Clara, CA, USA). Mouse PDAC cells were seeded in a 24-well plate (Agilent Technologies, 102342–100) at 1.5 × 10^4^ cells/well and incubated overnight. To eliminate the influence of cell proliferation capacity on the experiments, we performed all measurements of mouse PDAC cells within 12 h after seeding. The culture media consisted of DMEM (Agilent Technologies, 103575–100), glucose (1 M, Agilent Technologies, 103577–100), pyruvate (100 mM, Agilent Technologies, 103578–100), and L-glutamine (200 mM, Agilent Technologies, 103579–100). The OCR and ECAR (Agilent Technologies, 103015–100) were measured using sequential injections of oligomycin (1.5 μM), FCCP (1 μM), and Rot/AA (0.5 μM). For BM-DC measurements, Poly-D-Lysine solution (50 μg/mL, Sigma-Aldrich, A-003-E) was used to coat the 24-well plate (Agilent Technologies, 102342–100) at 4 °C overnight. Then, BM-DCs were seeded in the pre-coated 24-well plate (Agilent Technologies, 102342–100) at 4 × 10^5^ cells/well and centrifuged at 200 × g for 1 min. The control RPMI medium (Agilent Technologies, 103576–100) was supplemented with glucose (10 mM), pyruvate (1 mM), and L-glutamine (2 mM). The conditioned RPMI medium was supplemented with glucose (1 mM), pyruvate (1 mM), L-glutamine (2 mM), and L-LA (15 mM, Sigma-Aldrich, L7022-5G). The OCR and ECAR were measured using sequential injections of oligomycin (1.5 μM), FCCP (2 μM), and Rot/AA (0.5 μM). Each condition was performed with five replicates in a single experiment. Wave Desktop 2.6.3 software (Agilent Technologies) was used for data analysis.

### Analysis of single cell RNA sequencing (scRNA-seq) data

scRNA-seq analysis was performed as described previously [23].

#### Sample preparation

In brief, mouse PDAC cells (1 × 10^5^ shNC or shLDHA cells) were suspended in 50 µL PBS, then orthotopically injected into the pancreas of 5-week-old female C57BL/6 mice. After 20 days, the tumors were sliced into small pieces (2–4 mm) on ice. Single cell suspensions were acquired using the Tumor Dissociation Kit (Miltenyi Biotec, 130–096-730) at 37 °C for 40 min. One sample was integrated from five orthotopic syngeneic PDAC tumors. CD45^+^ immune cells in the tumor tissues were sorted by FCM, then cell suspensions were centrifuged and resuspended in PBS containing 0.2% BSA for scRNA-seq.

#### scRNA-seq library preparation and sequencing

The Chromium Next gel bead-in-emulsion (GEM) Single-cell 3' Reagent Kits v3.1 manual (10X Genomics, Pleasanton, CA, USA) was used to generate a library of 10^4^ cells/sample. The cDNA library was generated using a droplet-based sequencing platform (10X Genomics) and sequenced by DNBSEQ-G400 (MGI Tech, Shenzhen, China).

#### scRNA-seq data analysis

Raw sequencing data were aligned to the mm10 mouse reference genome and processed to a matrix containing normalized gene counts versus cells per sample by the Cell Ranger software package (version 5.0.0, 10X Genomics). The Seurat R package (version 4.3.0) was used to import and process the matrix. “CellCycleScoring”, “SCTransform”, and “Harmony (version 0.1.1)” were performed to eliminate any cell cycle influence or batch effects on the data [24]. The “DoubletFinder” R package (version 2.0.3) was used to filter the identified doublets [25]. The “RunPCA” function was used for principal component analysis and the “FindNeighbor” function was used to estimate clusters. Then, the “FindClusters” function by the Uniform Manifold Approximation and Projection (UMAP) method was used for visualization.

### Gene Ontology (GO) and Gene Set Enrichment Analysis (GSEA)

The GO (Metascape) and GSEA (ver. 4.3.2) analyses were performed on the genes upregulated in specific clusters (“FindAllMarkers” function) for pathway enrichment. The differentially expressed genes were analyzed using a two-sided Wilcoxon rank-sum test with Bonferroni FDR correction.

### Analysis of human PDAC scRNA-seq public datasets

Data analysis was performed using the methods described above. The scRNA-seq public datasets were obtained from the Genome Sequence Archive (GSA, CRA001160).

### Overall survival (OS) analysis

Human PDAC public datasets were obtained from The Cancer Genome Atlas (TCGA) database. Gene Expression Profiling Interactive Analysis (GEPIA) was used to analyze the mRNA expression levels of *LDHA*, *ITGAX* and *CD8A* for OS [26].

### Statistical analysis

The GraphPad Prism 9 software (GraphPad Software; San Diego, CA, USA) was used for statistical analysis. Data are represented as the mean ± standard deviation (SD). In vitro data represent one of three independent experiments. The unpaired Student’s t-test was used to analyze statistically significant differences between two groups. Survival analysis was performed using the Kaplan–Meier method and curves were compared by the log-rank test. The Wilcoxon rank-sum test was performed for scRNA-seq statistical analysis. Detailed information of the number of samples used in each experiment is included in the figure legends or methods. No statistical significance: ns *P* > 0.05. Statistical significance: * *P* < 0.05, ** *P* < 0.01, *** *P* < 0.001, **** *P* < 0.0001.

## Results

### PDAC exhibits a cold TME characterized by low-glucose and high-lactate

**Fig. 1:**
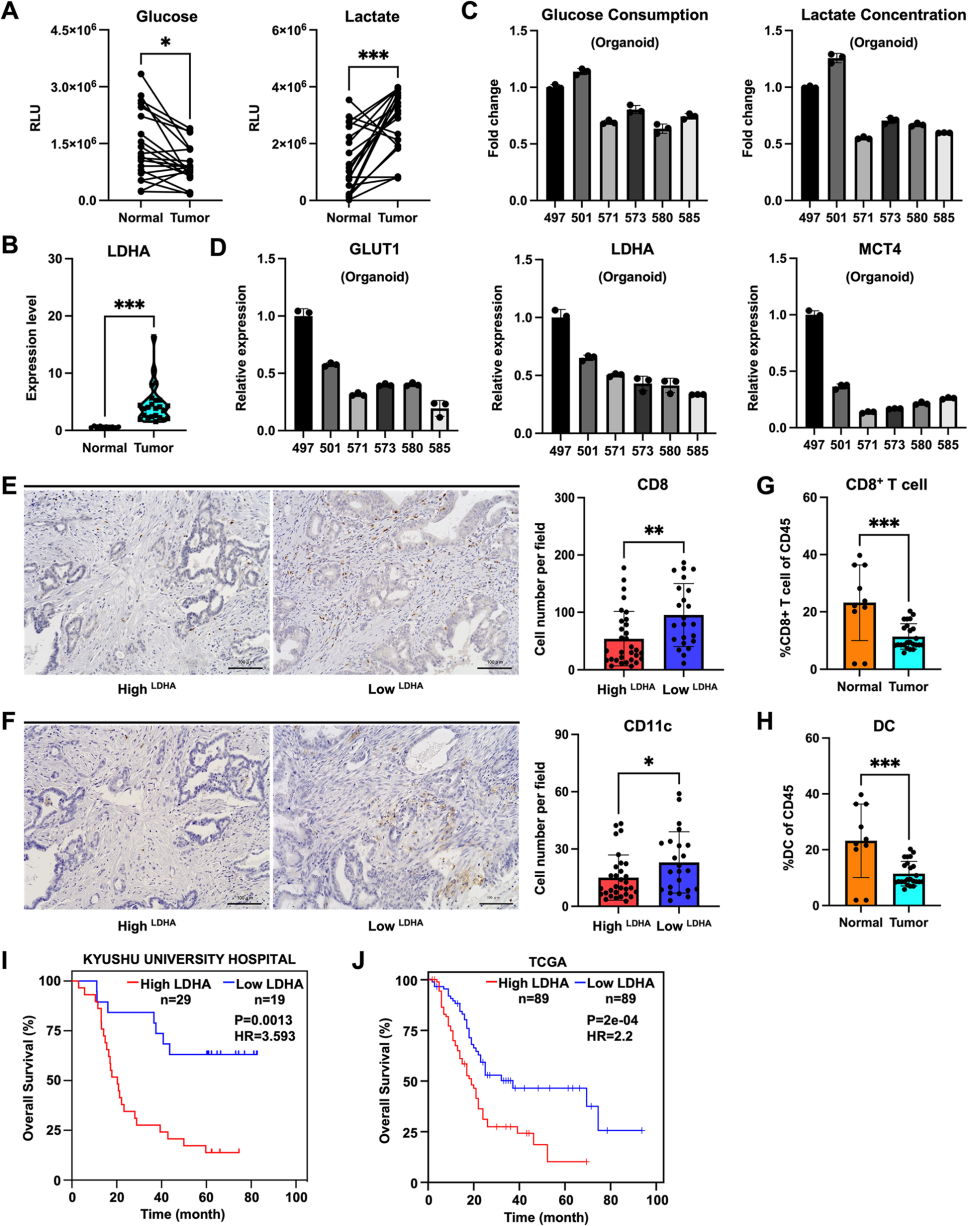
PDAC tumor glycolysis levels affect the numbers of DCs and CD8^+^ T cells. **A** Relative luminescence (RLU) levels of glucose and lactate in human PDAC tumors and paired adjacent normal pancreatic tissues (*n* = 20 patients). **B** We analyzed public scRNA-seq data and found that the relative *LDHA* expression levels were higher in cancer cells from primary pancreatic tumors of PDAC patients (Tumor) than those in normal pancreatic epithelial cells derived from individuals without PDAC (Normal). **C** Fold changes of glucose consumption and lactate production by PDOs. The results relative to Organoid 497 are shown (*n* = 3, 48 h). **D** qRT-PCR analysis of the mRNA expression levels of *GLUT1*, *LDHA*, and *MCT4* in PDOs relative to their expression levels in Organoid 497. The results were normalized to *β-ACTIN* mRNA expression levels (*n* = 3). **E**,** F** Representative IHC staining of (**E**) CD8^+^ cells and (**F**) CD11c^+^ cells in high LDHA PDACs (*n* = 29) and low LDHA PDACs (*n* = 19). Scale bar, 100 μm. **G**,** H** The analysis of the public PDAC scRNA-seq data revealed the proportions of (**G**) CD8^+^ T cells and (**H**) DCs among the CD45^+^ cells from normal pancreatic tissues of individuals without PDAC (Normal) and primary pancreatic tumors of PDAC patients (Tumor). **I**, **J** Overall survival analysis (Kaplan–Meier curve analysis) of PDAC patients, combining data from (**I**) Kyushu University Hospital (*n* = 29 high LDHA, *n* = 19 low LDHA) and (**J**) TCGA database (*n* = 89 high LDHA, *n* = 89 low LDHA)

We first measured the concentrations of glucose and lactate in paired PDAC tumor tissues and adjacent normal tissues (Fig. 1A). Compared with the adjacent normal tissues, the PDAC tumor tissues exhibited low glucose and high lactate concentrations. Cancer cells mainly consume glucose within the tumors, with LDHA being one of the key glycolysis-related enzymes [27]. We therefore analyzed publicly available PDAC scRNA-seq data [28] and observed elevated *LDHA* expression levels in cancer cells (Figs. 1B and S1A–C). To verify if the varying concentrations of glucose and lactate in tumors can be attributed to distinct cancer cell glycolysis activity, we examined the culture media of PDOs established in our laboratory [22]. Interestingly, PDOs of organoid 497 and 501 with increased glycolysis exhibited higher glucose consumption and more lactate production (Fig. 1C). Additionally, qRT-PCR analysis revealed corresponding changes in the mRNA expression levels of glycolysis-related genes in PDOs, including *GLUT1*, *LDHA*, and *MCT4* (Fig. 1D). Taken together, these results suggest that cancer cells with increased glycolysis can transform the PDAC TME into a low-glucose and high-lactate setting.

To assess the impact of varying glycolysis levels on immune cells in the TME, we performed IHC staining on pathological slides of PDAC patient samples corresponding to PDO. The results indicated a decreasing trend in the numbers of CD8^+^ cells (Figure S1D) and CD11c^+^ cells (Figure S1E) associated with increased glycolysis in PDOs. To more accurately evaluate the impact of increased glycolysis on immune cells, we expanded the number of pathological slides to 48 cases. Scoring and grouping were performed using the intensity of LDHA protein staining in the cancer cells (Figure S1F). Scores of 0^+^ and 1^+^ were defined as the low LDHA expression group and scores of 2^+^ and 3^+^ were defined as the high LDHA expression group. Subsequently, staining was performed for CD8 (Fig. 1E) and CD11c (Fig. 1F), then the positive cells were counted. The high LDHA expression group exhibited decreased numbers of CD8^+^ cells and CD11c^+^ cells, suggesting that increased glycolysis in cancer cells exerted an immunosuppressive effect on the TME. Analysis of the public PDAC scRNA-seq data also revealed that primary pancreatic tumors of PDAC patients with high *LDHA* expression levels showed decreased proportions of CD8^+^ T cells (Fig. 1G) and DCs (Fig. 1H) among CD45^+^ cells compared with normal pancreatic tissues of individuals without PDAC. Next, we analyzed PDAC patients and divided them into two groups: *LDHA*-high and *LDHA*-low expression in cancer cells. The results showed that DCs exhibited an enhanced antigen presentation gene significance score in the *LDHA*-low group compared with the *LDHA*-high group (Figure S1G and Table S2). Similarly, CD8^+^ T cells in the *LDHA*-low group showed higher Granzyme B (*GZMB*) and Perforin-1 (*PRF1*) expression levels than those in the *LDHA*-high group (Figure S1G). However, there were no significant differences in the proportion of DCs or CD8^+^ T cells among CD45^+^ cells between the two groups (Figure S1H).

OS analysis of our dataset indicated that patients in the high LDHA expression group showed shorter survival time (Fig. 1I). Similarly, analysis of TCGA data also showed that patients with high *LDHA* expression levels had a shorter survival time (Fig. 1J). We also conducted an OS analysis based on the CD11c and CD8 IHC staining data, as well as an analysis of the TCGA data. Both analyses showed no significant differences in OS between patients with high and low numbers of DCs (Figure S1I) or CD8^+^ T cells (Figure S1J). These results suggest that the glycolysis level in PDAC tumors can affect the composition of DCs and CD8^+^ T cells in the TME and lead to shorter OS.

### Impacts of cancer cells with increased glycolysis on the TME

**Fig. 2:**
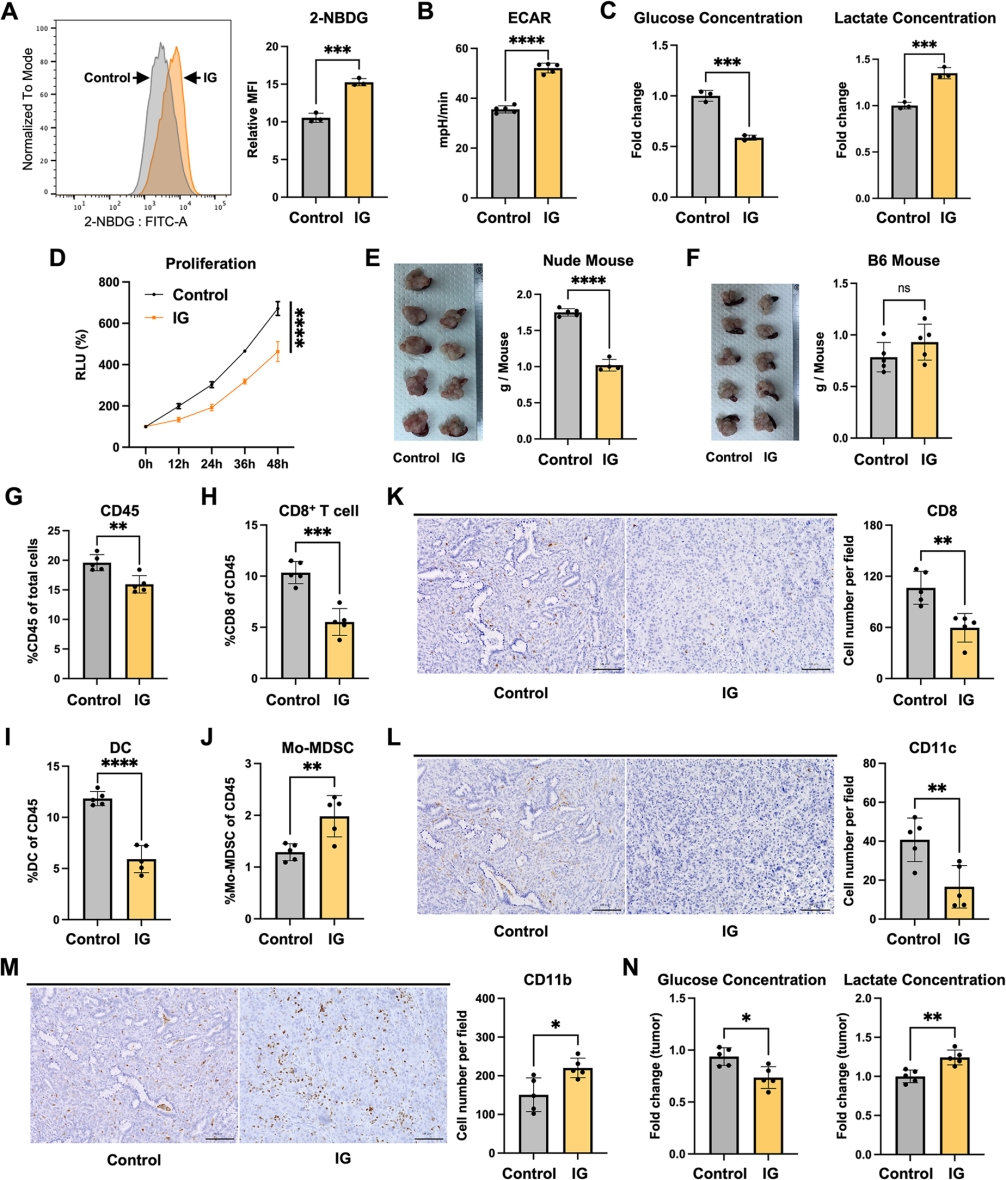
Increased glycolysis cells lead to the development of a low-glucose and high-lactate TME. **A** The FCM representative plot (left) and quantification (right) of 2-NBDG staining in control and increased glycolysis (IG) cells (*n* = 3). ‘Relative MFI’ denotes 2-NBDG MFI of stained samples relative to the matched unstained cells. **B** ECAR results of control and IG cells (*n* = 5). **C** Glucose consumption and lactate production of the control and IG cells (*n* = 3, 24 h). **D** Proliferation rates of the control and IG cells (*n* = 5). **E**, **F** Orthotopic transplantation of control and IG cells (*n* = 5) into (**E**) BALB/c-nu mice and (**F**) C57BL/6 mice for 18 days, followed by quantification of tumor weights (one of the BALB/c-nu mice transplanted with IG cells had died). **G**–**J** FCM analysis of (**G**) the percentages of tumor-infiltrating CD45^+^ cells in the control and IG tumors (*n* = 5) of C57BL/6 mice, and the percentages of (**H**) tumor-infiltrating CD8^+^ T cells, (**I**) DCs, and (**J**) Mo-MDSCs among the CD45^+^ cells. **K**–**M** Representative IHC staining of (**K**) CD8^+^ cells, (**L**) CD11c^+^ cells, and (**M**) CD11b^+^ cells in tumors of C57BL/6 mice. Scale bar, 100 μm. **N** Relative fold changes of glucose and lactate levels in the control and IG tumors (*n* = 5)

To investigate the impact of increased glycolysis on the TME using a mouse model, we established increased glycolysis (IG) cancer cells derived from KPC mice as described previously [12]. To verify the increased glycolytic capacity in cancer cells, we assessed 2-NBDG expression patterns using FCM (Fig. 2A) and glycolysis using an ECAR assay (Fig. 2B). Compared with the control cells, the IG cells exhibited enhanced glucose uptake and ECAR, indicating that the glycolysis levels increased in the IG cells. Furthermore, qRT-PCR analysis showed upregulation of the glycolysis-related *Hk2* and *Ldha* genes, as well as the major metabolic sensor *mTOR* gene, in the IG cells (Figure S2A). Similarly, measurements of the glucose and lactate concentrations in the culture media suggested that the IG cells exhibited higher glucose consumption and more lactate production (Fig. 2C). The cell migration (Figure S2B) and invasion (Figure S2C) rates were enhanced in the IG cells, indicating that increased glycolysis can possibly promote cancer cell malignancy. However, the IG cell proliferation capacity was weakened (Fig. 2D).

To explore the impact of increased glycolysis on immune cells, we performed orthotopic transplantation in both BALB/c-nu mice and C57BL/6 mice. We measured the tumor sizes 18 days after the transplantation. In BALB/c-nu mice, the control tumors were larger than the IG tumors, which was consistent with their proliferation capacity (Fig. 2E). However, in C57BL/6 mice, no significant difference in size was observed between the IG and control tumors (Fig. 2F), suggesting that the IG tumors displayed an enlargement trend. We hypothesized that increased glycolysis can possibly suppress the immune cell-mediated anti-tumor effects. Our FCM-based data of tumors transplanted in C57BL/6 mice showed a decreased number of CD45^+^ cells in the IG tumors (Figs. 2G and S2D), as well as lower proportions of CD8^+^ T cells (Fig. 2H), DCs (Fig. 2I), and CD4^+^ T cells (Figure S2G). In contrast, the proportion of monocytic myeloid-derived suppressor cells (Mo-MDSCs) was increased (Fig. 2J). However, no significant differences were observed in polymorphonuclear myeloid-derived suppressor cells (PMN-MDSCs) (Figure S2E) or tumor-associated macrophages (TAMs) (Figure S2F). Furthermore, IHC staining revealed that the numbers of CD8^+^ cells (Fig. 2K), CD11c^+^ cells (Fig. 2L), and CD4^+^ cells (Figure S2H) were decreased in the IG tumors, while the number of CD11b^+^ cells were increased (Fig. 2M). These IHC staining results were consistent with those of the FCM analysis. The αSMA and Sirius Red (SR) staining results showed increased proportions of αSMA- and SR-positive areas in IG tumors compared with control tumors (Figure S2I). Taken together, this suggests that cancer cells with increased glycolysis can affect the TME composition, including DCs, CD8^+^ T cells, and CAFs.

To verify if the increased glycolysis activity had also affected glucose and lactate levels, we measured the glucose and lactate concentrations in the tumors transplanted in C57BL/6 mice. Compared with the control tumors, the IG tumors exhibited a decreased glucose concentration and increased lactate concentration (Fig. 2N). In summary, these results suggest that increased glycolysis in IG cells can transform the TME of tumors into a low-glucose and high-lactate setting, leading to immunosuppression.

### Impacts of cancer cells with decreased glycolysis on the TME

**Fig. 3:**
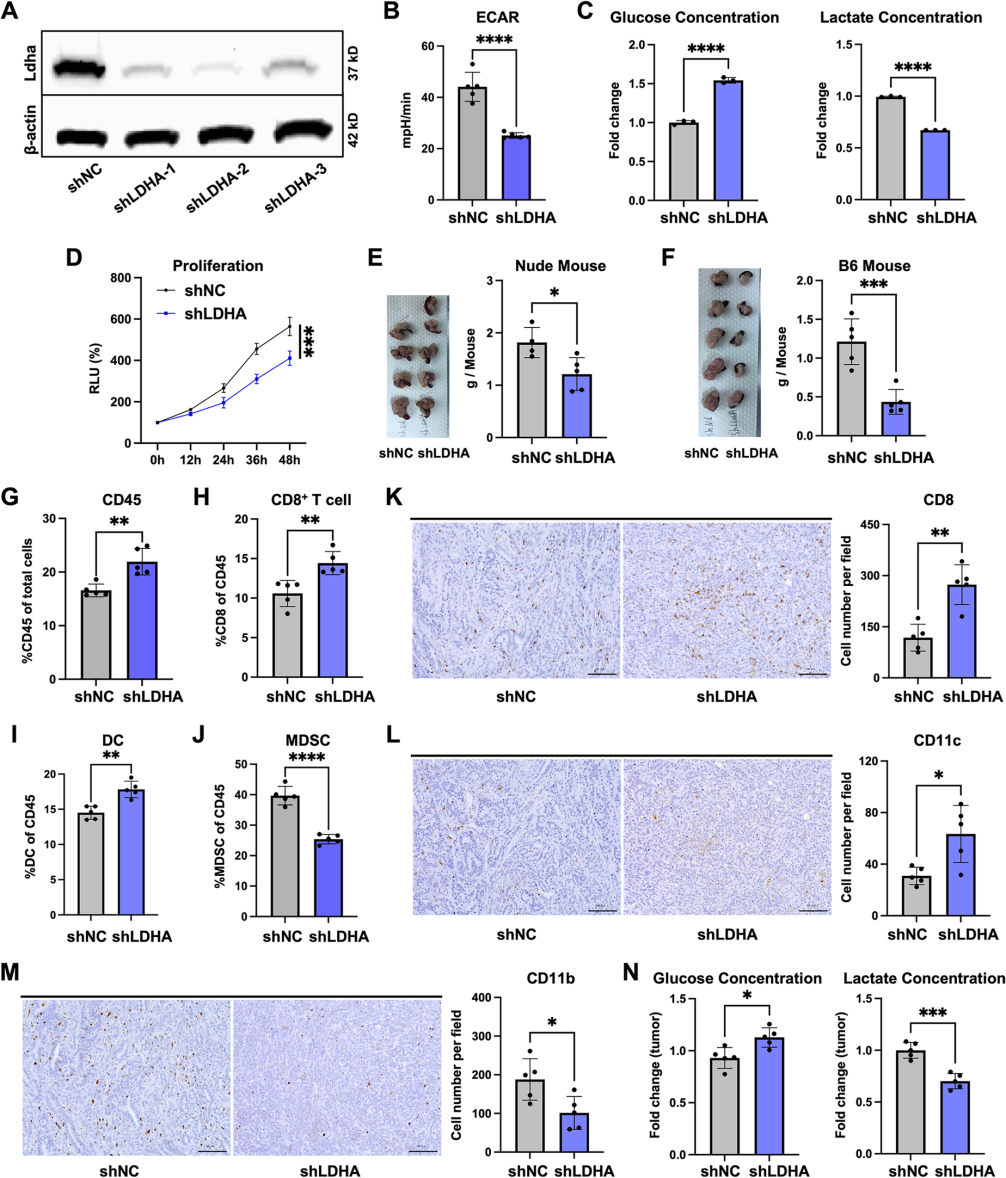
Decreased glycolysis cells lead to the development of a high-glucose and low-lactate TME.** A** Representative western blot analysis results of Ldha and β-actin protein levels in whole cell lysates of shNC, shLDHA-1, shLDHA-2, and shLDHA-3 cells. **B** ECAR results of shNC and shLDHA cells (*n* = 5). **C** Glucose consumption and lactate production by shNC and shLDHA cells (*n* = 3, 24 h). **D** Proliferation rates of shNC and shLDHA cells (*n* = 5). **E**, **F** Orthotopic transplantation of shNC and shLDHA cells (*n* = 5) into (**E**) BALB/c-nu mice and (**F**) C57BL/6 mice for 20 days, followed by quantification of tumor weight (one of the BALB/c-nu mice transplanted with shNC cells had died). **G**–**J** FCM analysis of (**G**) the percentages of tumor-infiltrating CD45^+^ cells in shNC and shLDHA tumors (*n* = 5) of C57BL/6 mice, and the percentages of (**H**) tumor-infiltrating CD8^+^ T cells, (**I)** DCs, and (**J**) MDSCs among the CD45^+^ cells. **K**–**M** Representative IHC staining of (**K**) CD8^+^ cells, (**L**) CD11c^+^ cells, and (**M**) CD11b^+^ cells in tumors of C57BL/6 mice. Scale bar, 100 μm. **N** Relative fold changes of glucose and lactate levels in shNC and shLDHA tumors (*n* = 5)

Because increased glycolysis in cancer cells can contribute to an immunosuppressive TME, we aimed to explore if inhibiting cancer cell glycolysis could enhance the anti-tumor potential of immune cells. In this study, we established shLDHA cells with decreased glycolysis [27]. The efficiency of *Ldha* gene knockdown was validated using western blot (Fig. 3A) and qRT-PCR (Figure S3A) analyses. Of the shRNAs tested, shLDHA-2 exhibited the highest knockdown efficiency and was therefore selected for subsequent experiments. To verify the decreased glycolytic capacity in cancer cells, we assessed 2-NBDG expression by FCM (Figure S3B) and glycolysis with ECAR assays (Fig. 3B). Compared with shNC cells, shLDHA cells exhibited the effects of weakened glucose uptake, with the ECAR results revealing decreased glycolysis levels in shLDHA cells. Furthermore, shLDHA cells showed lower glucose consumption and lactate production (Fig. 3C). Additionally, the shLDHA cell migration (Figure S3C), invasion (Figure S3D), and proliferation (Fig. 3D) rates were reduced, indicating that decreased glycolysis can inhibit the malignant potential of cancer cells.

To explore the impact of decreased glycolysis on immune cells, we performed orthotopic transplantation in both BALB/c-nu and C57BL/6 mice. We observed a reduced shLDHA tumor size 20 days post-transplantation in both BALB/c-nu mice (Fig. 3E) and C57BL/6 mice (Fig. 3F), which was consistent with their cell proliferation capacity. However, the reduction in tumor size was more significant in C57BL/6 mice than in BALB/c-nu mice, suggesting that decreased glycolysis in cancer cells may lead to an enhanced immune cell-mediated anti-tumor response. To investigate the impacts of cancer cells with decreased glycolysis on immune cells, we performed FCM analysis of tumors transplanted in C57BL/6 mice. The number of CD45^+^ cells was increased in shLDHA tumors (Fig. 3G), as well as the proportions of CD8^+^ T cells (Fig. 3H), DCs (Fig. 3I), TAMs (Figure S3F), and CD4^+^ T cells (Figure S3G) among the total CD45^+^ cells. However, the proportions of MDSCs (Fig. 3J) and PMN-MDSCs (Figure S3E) were decreased. Furthermore, IHC staining revealed that the numbers of CD8^+^ cells (Fig. 3K), CD11c^+^ cells (Fig. 3L), and CD4^+^ cells (Figure S3H) were increased in shLDHA tumors, while the number of CD11b^+^ cells was decreased (Fig. 3M). The IHC results were consistent with those of the FCM analysis. Compared with shNC tumors, shLDHA tumors showed reduced proportions of αSMA- and SR-positive areas (Figure S3I). Taken together, this suggests that cancer cells with decreased glycolysis can affect the TME composition, including DCs, CD8^+^ T cells, and CAFs.

We next examined if the decreased glycolysis activity had altered the glucose and lactate levels in the tumors transplanted in the C57BL/6 mice. Compared with the shNC tumors, the shLDHA tumors exhibited an increased glucose concentration and decreased lactate concentration (Fig. 3N). In summary, these data suggest that decreased shLDHA cell glycolysis can lead to a high-glucose and low-lactate TME, resulting in increased anti-tumor immunity.

### Analysis of DC and CD8^+^T cell functions by scRNA-seq in a high-glucose and low-lactate TME

**Fig. 4:**
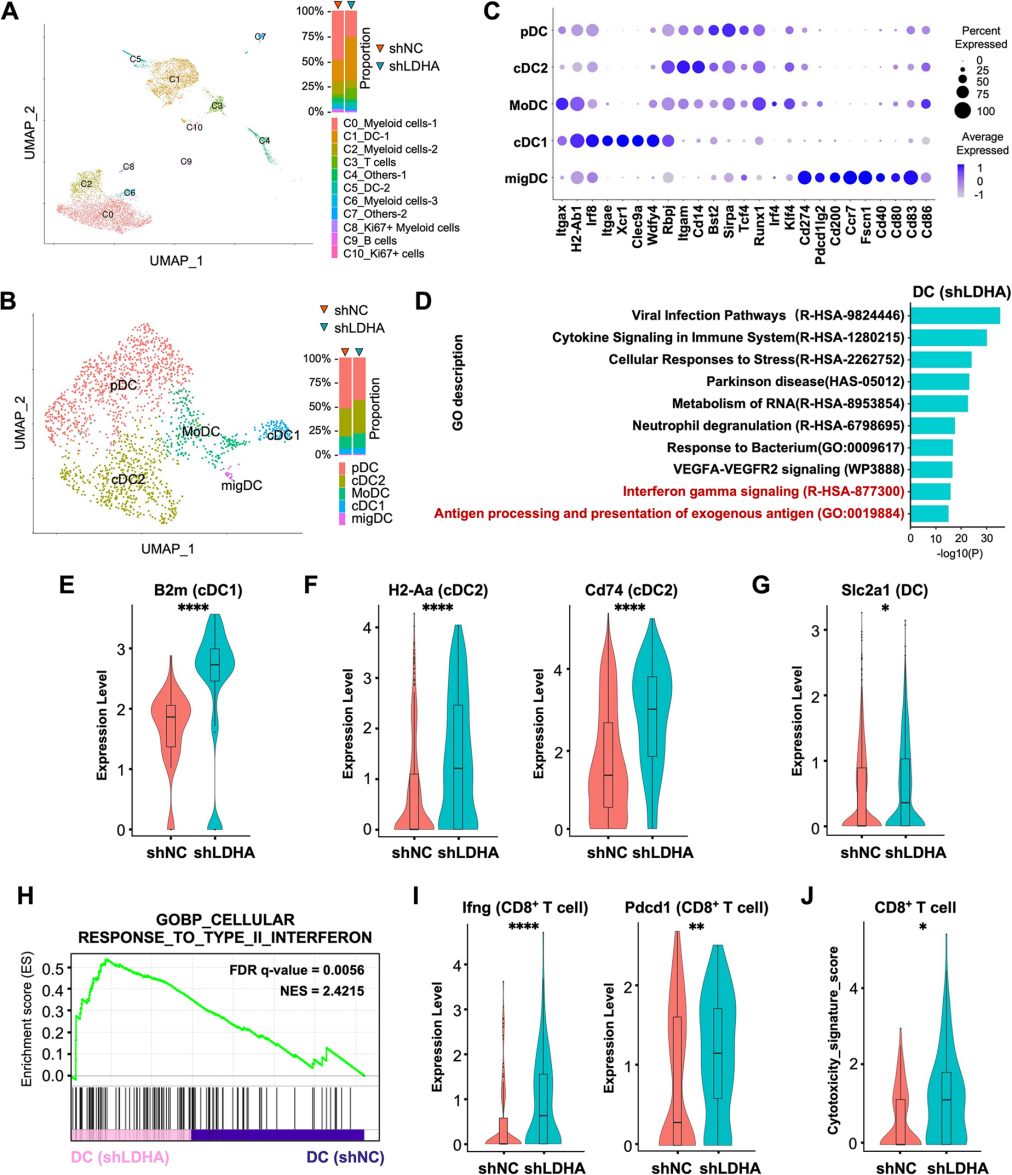
High-glucose and low-lactate conditions promote the DC antigen-presenting function and enhance CD8^+^ T cell activity. **A** The UMAP plots of 5,393 CD45^+^ cells (shNC tumors, *n* = 5) and 1,974 CD45^+^ cells (shLDHA tumors, *n* = 5). CD45^+^ cells were classified into 11 subsets using known marker genes. **B** The UMAP plots of 1,125 DCs (shNC tumors) and 712 DCs (shLDHA tumors) were classified into four subsets using known marker genes. **C** The dot plots of representative genes related to DC subsets and Z-scores normalized log2. The center represents the average expression level across all single cells with a color scale from 1 to -1. **D** Gene Ontology (GO) terms associated with the differentially expressed genes between DCs from shLDHA tumors and shNC tumors (analyzed using Metascape). **E**–**G** Violin plots showing the relative expression levels of (**E**) B2m in cDC1, (**F**) H2-Aa and CD74 in cDC2, and (**G**) Sl2ac1 in DCs. **H** The GSEA results showing the enrichment of RESPONSE_TO_TYPE_II_INTERFERON in DCs. **I**,** J** Violin plots showing (**I**) the relative expression levels of IFN-γ and PDCD1 in CD8^+^ T cells and (**J**) the cytotoxicity signature scores of CD8^+^ T cells

FCM data of orthotopically transplanted tumors in C57BL/6 mice revealed that the proportions of DCs and CD8^+^ T cells were decreased in IG tumors, whereas the proportions of DCs and CD8^+^ T cells were increased in shLDHA tumors. Although these data suggested that glycolysis levels could influence the immune cell composition within the TME, the specific impacts on their functions had not yet been explored. To better understand this, we performed scRNA-seq analysis on the CD45^+^ cells isolated from shNC and shLDHA tumors. Furthermore, to reduce the impact of tumor heterogeneity, the samples of shNC and shLDHA were integrated from 5 tumors, respectively. The CD45^+^ cells were classified into 11 clusters as C0 (Myeloid cells-1), C1 (DC-1), C2 (Myeloid cells-2), C3 (T cells), C4 (Others-1), C5 (DC-2), C6 (Myeloid cells-3), C7 (Others-2), C8 (Ki67^+^ Myeloid cells), C9 (B cells), and C10 (Ki67^+^ cells) using known marker genes [29, 30], with the batch effects accurately removed through integration using ‘Harmony’ (Figs. 4A and S4A–C). The proportions of DCs in C1 (DC-1) and T cells in C3 (T cells) were increased, corroborating the FCM analysis results. Subsequently, to focus on DCs, C1 (DC-1) and C5 (DC-2) were further divided into five subsets as plasmacytoid DCs (pDCs), cDC1s, cDC2s, monocyte-derived DCs (MoDCs) and migratory DCs (migDCs) using known marker genes (Figs. 4B and S4D). The expression levels of relevant genes are also shown in Dot plots (Fig. 4C). Because there were no significant changes in the proportions of DC subsets, we then proceeded to analyze their functions.

Next, we performed GO pathway analysis of the genes with increased expression levels in DCs from shLDHA tumors (Table S1). This demonstrated that the interferon gamma signaling pathway and antigen processing and presentation of exogenous antigen pathway were enhanced in these tumors (Fig. 4D). The violin plots revealed increased relative expression of both the major histocompatibility complex class I (MHC I)-related gene B2m in cDC1 (Fig. 4E) and the MHC II-related genes H2-Aa and Cd74 in cDC2 (Fig. 4F) in shLDHA tumors. Furthermore, the increased expression of Slc2a1 in DCs from shLDHA tumors (Fig. 4G) suggested an enhanced glucose uptake capacity, potentially contributing to improved mitochondrial oxidative phosphorylation (OXPHOS). Taken together, these data suggest that the antigen presentation function and glucose uptake capacity of DCs were increased in shLDHA tumors with high glucose and low lactate concentrations.

GSEA showed an increased “RESPONSE TO TYPE II INTERFERON” of DCs in shLDHA tumors (Fig. 4H and Table S1)). We considered that interferon gamma (IFN-γ) secreted by CD8^+^ T cells was potentially increased in tumors with high glucose and low lactate concentrations, which could further enhance the DC antigen-presenting function. Next, we subdivided the C3 (T cells) into two subsets, CD8^+^ T cells and CD4^+^ T cells (Figures S4E and S4F), and analyzed the CD8^+^ T cell subsets. As hypothesized, there were increased INF-γ and Pdcd1 expression levels in CD8^+^ T cells, which suggested enhanced T cell activation (Fig. 4I). The cytotoxicity signature score (Fig. 4J and Table S2) was improved, while the expression levels of tricarboxylic acid (TCA) signature score (Table S2) and Slc2a3 were also upregulated (Figures S4G and S4H). Taken together, a high-glucose and low-lactate TME resulted in enhanced DC antigen presentation, facilitating the activation of CD8^+^ T cells. Subsequently, the activated CD8^+^ T cells secreted INF-γ, further promoting DC function and thereby facilitating immune cycling.

### Effects of cancer cell glycolysis levels on DC functions

**Fig. 5:**
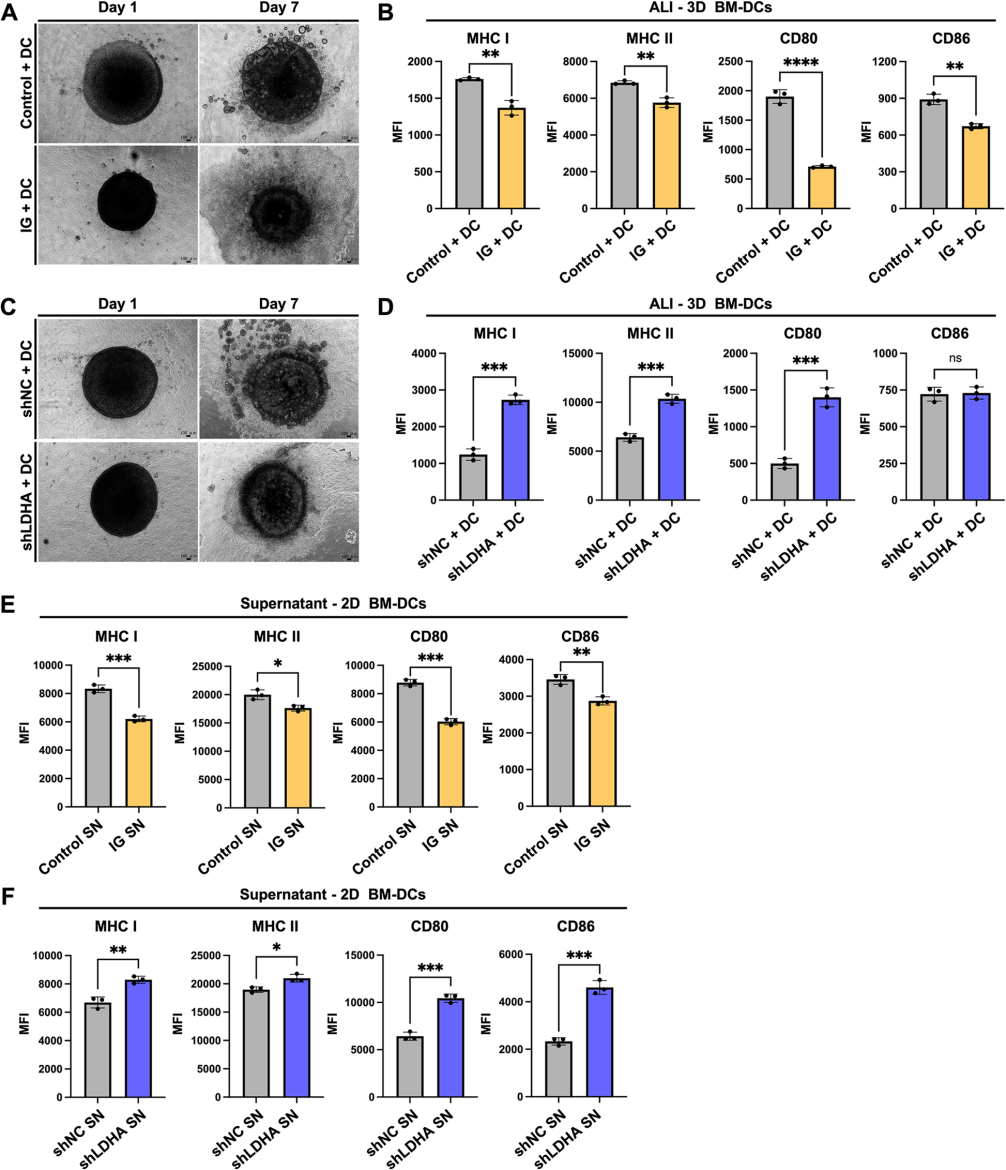
Cancer cells with increased glycolysis can inhibit the antigen-presenting function of BM-DCs.** A** Representative images of BM-DCs co-cultured with control and IG cells (*n* = 3), respectively, using ALI-3D co-culture. **B** FCM analysis of the MHC I, MHC II, CD80, and CD86 expression levels in BM-DCs. **C** Representative images of BM-DCs co-cultured with shNC and shLDHA cells (*n* = 3), respectively, using ALI-3D co-culture. **D** FCM analysis of the MHC I, MHC II, CD80, and CD86 expression levels in BM-DCs. After 2D culture of cancer cell supernatant and BM-DCs for 48 h, the MHC I, MHC II, CD80, and CD86 expression levels of BM-DCs were analyzed by FCM. **E** Control and IG supernatants (*n* = 3). **F** shNC and shLDHA supernatants (*n* = 3)

To confirm the results of the scRNA-seq analysis of orthotopically transplanted tumors in C57BL/6 mice, we performed in vitro experiments. Because many previous reports have described the impacts of glycolysis on T cells [11, 12, 31], we focused on the antigen-presenting function of DCs through in vitro co-culture experiments in this study. Because 3D cell culture can better recapitulate tumor metabolism and cell signaling [32], we established an ALI-3D co-culture model (Figure S5A) by combining methods from previous studies [20, 21] to simulate the TME. Additionally, we induced BM-DCs with a predominance of cDC1 from C57BL/6 mice [19]. This model enabled prolonged co-culture of cancer cells and BM-DCs for over a week. On Day 7, we analyzed BM-DCs in ALI organoids by FCM (Figure S5B). The representative images show BM-DCs co-cultured with control and IG cells (Fig. 5A). Compared with the control cells, BM-DCs co-cultured with IG cells decreased the expression levels of not only MHC I and MHC II, but also of CD80 and CD86 (Figs. 5B and S5C). The representative images show BM-DCs co-cultured with shNC and shLDHA cells (Fig. 5C). Furthermore, compared with BM-DCs co-cultured with shNC cells, those co-cultured with shLDHA cells increased the expression levels of MHC I and MHC II, as well as of CD80 (Figs. 5D and S5D). These results were consistent with those of the scRNA-seq analysis of DCs in tumors, suggesting that the DC antigen-presenting function was enhanced in a high-glucose and low-lactate TME.

As shown in Figs. 2C and 3C, cancer cells with distinct glycolysis levels consumed varying amounts of glucose and produced different lactate concentrations in the culture media. To investigate if this antigen-presenting function could be directly attributed to cancer cell activity or was indirectly influenced by varying glucose and lactate concentrations, we performed co-culture experiments using cancer cell supernatants and BM-DCs in vitro. Previous studies have indicated that DCs predominantly use intracellular glycogen during the initial 24 h of nutrient-deprived conditions [33], followed by nutrient consumption from the culture media. Consequently, we extended the co-culture time to 48 h. The FCM analysis indicated that BM-DCs cultured in the IG supernatant exhibited reduced expression levels of MHC I, MHC II, CD80, and CD86 (Figs. 5E, S5E and S6B), consistent with our observations using ALI-3D co-culture with IG cells. Conversely, BM-DCs cultured in the shLDHA supernatant showed increased expression levels of MHC I, MHC II, CD80, and CD86 (Figs. 5F and S5F), consistent with the findings of ALI-3D co-culture with shLDHA cells. Therefore, the impact on the BM-DC antigen-presenting function in this experiment was not from a direct effect of the cancer cells, but rather from an indirect effect of different glucose and lactate concentrations in the supernatant.

In summary, our in vitro findings indicate that the increased glycolysis of cancer cells can inhibit the antigen-presenting function of DCs.

### Low-glucose and high-lactate can inhibit the DC antigen-presenting function by attenuating mitochondrial OXPHOS

**Fig. 6:**
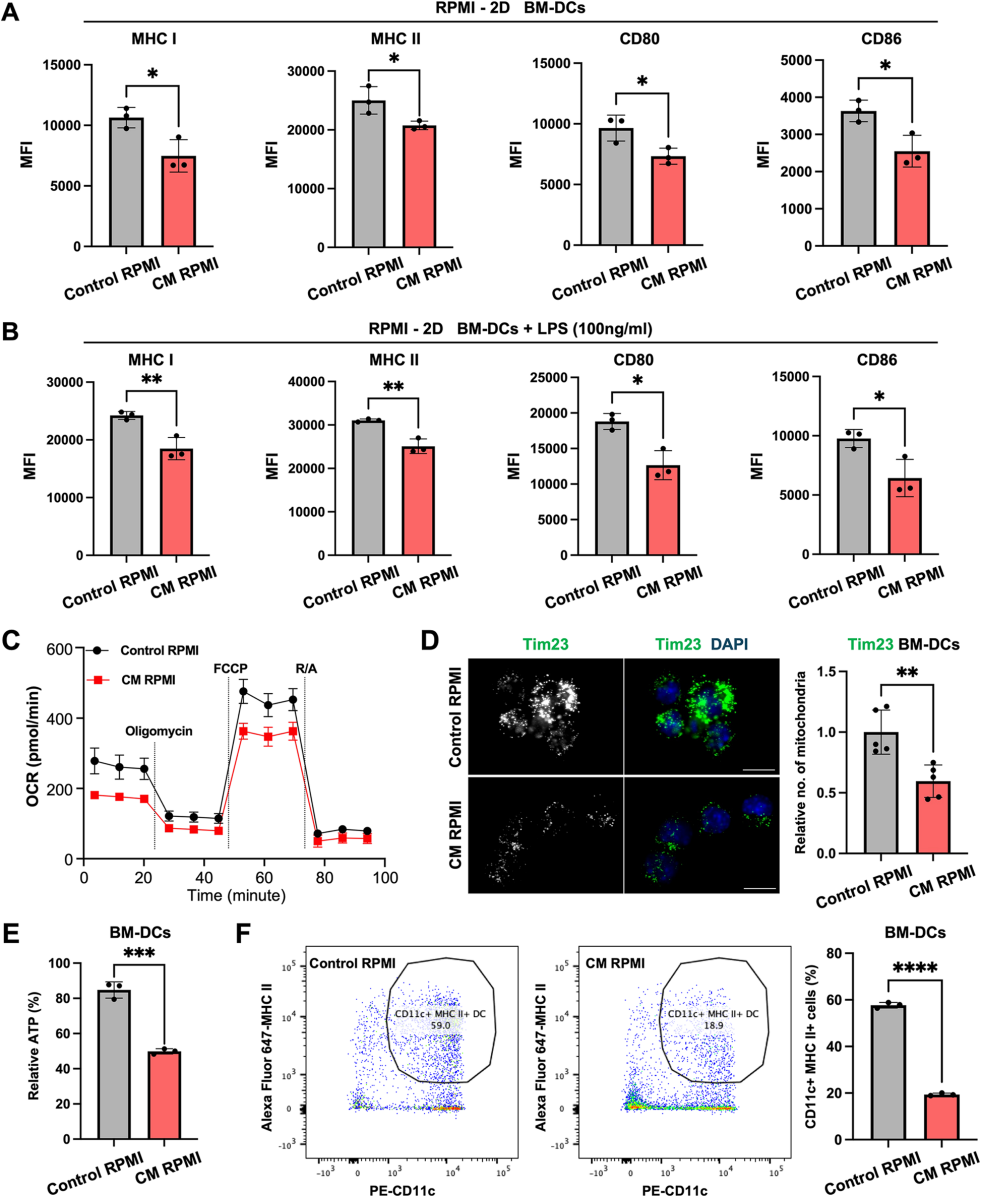
The BM-DC antigen-presenting function is inhibited by mitochondrial OXPHOS dampening in low-glucose and high-lactate conditions. **A**, **B** BM-DCs were cultured in control RPMI and CM RPMI for 48 h, and the expression levels of MHC I, MHC II, CD80 and CD86 were analyzed by FCM, (**A**) without LPS (**A**) and (**B**) with 100 ng/mL LPS. Control RPMI (11 mM glucose and 0 mM lactate) and conditioned media (CM) RPMI (1 mM glucose and 15 mM lactate).** C** Real-time analysis of the OCR of pre-cultured BM-DCs in control and CM RPMI for 48 h (*n* = 5).** D** Representative mitochondria (TIM23) images of pre-treatment BM-DCs in control and CM RPMI for 48 h (left) and the quantification of the number of mitochondria (right) (*n* = 5). **E** Relative ATP levels of BM-DCs in control and CM RPMI (*n* = 3, 48 h). **F** Representative images (left) and the percentages of BM-DC (CD11c^+^ MHC II^+^) differentiation rates (right) in control and CM RPMI (*n* = 3, 9 days)

Research has demonstrated that cancer cells not only consume nutrients in the culture media, but also secrete various cytokines. To focus on the influence of glucose and lactate on the DC antigen-presenting function, we directly adjusted their concentrations in RPMI 1640 media. Following the glucose and lactate concentrations used in published T cell research [34], we selected the conditioned media (CM) RPMI with 1 mM glucose and 15 mM lactate for in vitro experiments. After 48 h of culture, compared with levels with control RPMI, the MHC I, MHC II, CD80, and CD86 expression levels in BM-DCs were decreased with CM RPMI (Figs. 6A, S6B and S6C). However, changes were observed in the absence of Toll-like receptor (TLR) stimulation. To examine this, we used lipopolysaccharide (LPS) to stimulate TLR4 and evaluated any effects on the antigen-presenting function and costimulatory molecule expression. Compared with the levels with control RPMI, the reduced expression levels of MHC I, MHC II, CD80, and CD86 were further decreased with CM RPMI (Figs. 6B and S6D). Overall, these results indicate that low glucose and high lactate levels had inhibitory effects on the antigen-presenting function of BM-DCs.

Considering that insufficient nutrients may affect OXPHOS and ATP production DCs cultured in low-glucose and high-lactate conditions, we examined the function of mitochondria, the primary cellular organelle for aerobic glycolysis. BM-DCs were pre-cultured in control and CM RPMI for 48 h, with the OCR results showing that mitochondrial OXPHOS in BM-DCs was suppressed in CM RPMI compared with that in the control RPMI (Fig. 6C). Additionally, the IF staining results revealed that mitochondria were reduced in BM-DCs in CM RPMI compared with control RPMI (Figs. 6D and S6A). ATP assays also showed that the intracellular ATP levels of BM-DCs were reduced with CM RPMI compared with the control RPMI (Fig. 6E). Overall, the low-glucose and high-lactate conditions can inhibit the aerobic respiration of mitochondria in BM-DCs, leading to a reduction of ATP generated through aerobic glycolysis and ultimately resulting in a weakened antigen-presenting function. We also simultaneously investigated the impact of CM RPMI on BM cell differentiation into BM-DCs, observing that the differentiation rates were significantly reduced (Fig. 6F). This was therefore a potential reason for the reduced number of DCs in the TME.

In summary, our data suggest that the DC antigen-presenting function was inhibited and their numbers were reduced in a low-glucose and high-lactate TME.

## Discussion

PDAC exhibits metabolic characteristics that can promote tumor invasion and treatment resistance, which can possibly be used to develop new treatment methods [35, 36]. To date, the impacts of glycolytic alterations in PDAC on the TME remain poorly understood. In this study, we initially examined variations in glucose and lactate concentrations within PDAC tumor tissues, observing that the concentrations were diverse among different tumors. Subsequently, the results of experiments evaluating PDO glycolysis levels showed differences among cancer cells. Furthermore, IHC staining indicated reduced numbers of DCs and CD8^+^ T cells in samples with high *LDHA* expression levels, suggesting that the PDAC glycolytic level could affect the TME. Therefore, we conducted further studies using orthotopic transplantation mouse models. In tumors with increased glycolysis, the TME was characterized by low glucose and high lactate levels, decreased numbers of DCs and CD8^+^ T cells, and a higher number of MDSCs. Conversely, tumors with decreased glycolysis exhibited the opposite changes. The results of in vitro and scRNA-seq analysis showed that under low-glucose and high-lactate conditions, DC mitochondrial aerobic respiration was inhibited, leading to a weakened antigen-presenting function. This indicated that inhibiting glycolysis in PDAC could enhance the antigen-presenting function of DCs, thereby facilitating immune cycling.

Metabolic reprogramming is a hallmark of cancer cells, characterized by transitioning mitochondrial OXPHOS to anaerobic glycolysis. Glucose uptake and lactate generation rates are typically high in tumor tissues, which may result in reduced extracellular glucose levels and increased lactate levels [37, 38]. LDHA, as the final enzyme in the glycolytic pathway, plays a crucial role in PDAC tumor growth and metastasis, impacting the number of MDSCs and the anti-tumor capacity of natural killer (NK) cells in the TME [39]. In our findings, *Ldha* knockdown resulted in a TME characterized by high-glucose and low-lactate, enhancing the activity of DCs and T cells. Previous studies have examined the tumor interstitial fluid and serum in humans and mice, revealing an insufficient supply of metabolic substrates to tumor cells and an increased abundance of other metabolites [40, 41]. Here, we measured the glucose and lactate concentrations in human tumor tissues and adjacent normal tissues. Compared with these adjacent normal tissues, the tumors were characterized by a low-glucose and high-lactate microenvironment. Experiments using the orthotopic transplantation of mouse PDAC cells showed that tumors with increased glycolysis exhibited a low-glucose and high-lactate TME, indicating that the glycolytic capacity of cancer cells could influence the composition of both nutrients and immune cells in the TME. In our study, we observed that mouse PDAC cells with increased glycolytic activity exhibit enhanced invasive and metastatic capabilities. However, further studies are needed to determine whether the glucose and lactate properties in the primary TME extend to the corresponding metastatic sites.

Lactate accumulation in the TME has been shown to inhibit the differentiation of monocytes and DCs [42]. In this current work, we observed a reduced efficiency in inducing BM differentiation into DCs under low-glucose and high-lactate conditions. Suppressing glycolysis using glucose-deficient culture media or the glycolysis inhibitor 2-deoxyglucose was previously reported to reduce the expression levels of activation markers (such as CD80 and CD86) in BM-derived DCs, as well as lower pro-inflammatory cytokine secretion [43, 44]. In other cancer types, tumor-derived lactate can restrict DC presentation of tumor-specific antigens to other immune cells. Additionally, pDC function is impaired by lactic acidosis in melanoma patients, which has also been observed in breast cancer patients and mouse models [45–47]. The effect of pH on antigen uptake can be either negative or positive, depending on the receptor binding to the antigen and if the antigen-MHC-I complex prefers a neutral environment [48]. Previous studies have shown that low-glucose or high-lactate conditions has an inhibitory effect on the antigen-presenting function of MoDCs or pan-DCs induced by GM-CSF and IL-4. In our experiment, however, the FLT3-L differentiation method was used to induce BM-DCs, mainly focusing on cDC1. To better simulate TME conditions with in vitro experiments and avoid the influence of low pH on BM-DCs, we used culture media with 1 mM glucose and 15 mM L-LA to evaluate the impact on BM-DC antigen-presenting function.

According to previous studies, DCs use pre-existing stored glycogen to support the switch from OXPHOS to glycolysis during inflammatory stimulation [33]. TLR agonists (such as LPS) induce DC activation, accompanied by increased glycolysis activity and the elimination of ATP-coupled mitochondrial respiration, with a corresponding decrease in OXPHOS [49]. In this study, we aimed to reduce the impact of intracellular glycogen on DCs by increasing the time of culture in a low-glucose and high-lactate environment. We combined the methods of previous organoid co-culture models and ALI organoid models, creating a novel ALI-3D co-culture model of cancer cells and BM-DCs. In this model, BM-DCs could survive for over a week, supporting better observations of the interactions between cancer cells and BM-DCs. Using both 2D and 3D culture methods, we confirmed the simultaneous effects of glucose and lactate on the BM-DC antigen-presenting function.

Despite an increased understanding of the dynamic nature of PDAC metabolism over the past decade, this knowledge has not yet been effectively translated into clinically relevant therapeutic intervention methods. In this study, we conducted in vivo and in vitro experiments using induced high glycolysis cancer cells and low glycolysis cancer cells with *Ldha* knockdown. Previous research has also used experiments with LDHA inhibitors, enhancing the anti-tumor activity of CD8^+^ T cells [50]. However, there remains a lack of cancer cell-specific glycolysis inhibitors in clinical applications. In future PDAC studies, it is imperative to employ various models of glycolysis augmentation and reduction. Experiments can be conducted using genetically engineered KPC mice with enhanced and impaired glycolysis, allowing any changes in cancer cells and immune cells under different glucose and lactate concentrations to be observed. This approach would aim to identify specific therapeutic targets in cancer cells to inhibit glycolysis, while simultaneously ensuring minimal impacts to immune cells. In this study, we only evaluated the effects of glucose and lactate on mouse-derived DCs. Future research should evaluate the effects on human-derived DCs. To better elucidate the detailed mechanisms of weakened DC antigen presentation function in low-glucose and high-lactate environments, we plan to examine the chromatin of DCs in subsequent studies, clarifying any changes in relevant genes.

In conclusion, we found that alterations in cancer cell glycolysis in PDAC can influence the immune cell composition of the PDAC TME. In a low-glucose and high-lactate environment, DCs had a reduced number and weakened antigen-presenting function. Metabolic reprogramming driven by KRAS mutations in PDAC significantly affect the TME [5], especially the function of immune cells such as CD8 + T cells. Furthermore, novel KrasG12D inhibitors, such as MRTX1133, can possibly regulate metabolic pathways like glycolysis, as well as modulate the immune landscape [51, 52]. Such targeted therapy focusing on PDAC glycolysis, in conjunction with relevant immunotherapies and chemotherapy, may potentially achieve the goal of fully eliminating these tumors.

## Conclusions

This study demonstrates that increased glycolysis in PDAC tumors results in a low-glucose, high-lactate tumor microenvironment (TME), which suppresses immune cell function, particularly dendritic cells (DCs) and CD8 + T cells. This glycolytic shift leads to reduced antigen presentation by DCs and decreased anti-tumor immunity, contributing to poorer patient survival. Conversely, inhibiting glycolysis enhances glucose availability and reduces lactate levels, thereby boosting DC and CD8 + T cell function and improving anti-tumor responses. These findings highlight the potential of targeting tumor metabolism to enhance immunotherapy effectiveness in PDAC.

## Supplementary Information

Supplementary Material 1: Figure S1. Public scRNA-seq data analysis and IHC staining. (A) We analyzed public scRNA-seq data and found that the UMAP plots of 163,830 cells from primary pancreatic tumors of PDAC patients (Tumor) and normal pancreatic tissues of patients without PDAC (Normal) were classified into nine subsets using known marker genes. (B) Violin plots showing the relative expression levels of marker genes with high differential expression among the nine clusters. (C) UMAP plots showing the expression levels of representative genes in the nine clusters. (D, E) Representative IHC staining of (D) CD8^+^ cells and (E) CD11c^+^ cells corresponding to PDOs. Scale bar, 100 μm. (F) LDHA expression IHC scoring levels in PDAC samples: 0^+^ and 1^+^ scores are the low LDHA expression group and 2^+^ and 3^+^ scores are the high LDHA expression group. (G) Analysis of public scRNA-seq data revealed an increased antigen presentation gene significance score in DCs, as well as higher *GZMB* and *PRF1* expression levels, in CD8^+^ T cells from primary pancreatic tumors of PDAC patients with low *LDHA* expression levels (Low LDHA) compared with those from PDAC tumors with high *LDHA* expression levels (High LDHA). (H) Analysis of public scRNA-seq data revealed no significant difference in the proportion of DCs or CD8^+^ T cells among the CD45^+^ cells between PDAC tumors with low *LDHA* expression levels (Low LDHA) and high *LDHA* expression levels (High LDHA). (I, J) OS analyses (Kaplan–Meier curve analysis) of PDAC patients were performed based on the expression levels of (I) CD11c (*ITGAX*) and (J) CD8 (*CD8A*), using both our cohort data and TCGA datasets.

Supplementary Material 2: Figure S2. Increased glycolysis can enhance the migration and invasion of cancer cells. (A) qRT-PCR analysis of the *Hk2*, *Ldha* and *mTOR* mRNA expression levels in control and increased glycolysis (IG) cells. The expression levels are relative to those in the control cells and were normalized to *β-actin* mRNA expression (*n* = 3). (B, C) Representative images of control and IG cell hematoxylin and eosin (H&E) staining, and the number of (B) migrating and (C) invading cells (*n* = 3, migration for 24 h and invasion for 48 h). (D) Representative gating strategies for tumor-infiltrating DCs, PMN-MDSCs, Mo-MDSCs, and TAMs. (E–G) FCM analysis of the percentages of tumor-infiltrating (E) PMN-MDSCs, (F) TAMs, and (G) CD4^+^ T cells among the CD45^+^ cells of control and IG tumors (*n* = 5) from C57BL/6 mice. (H) Representative IHC staining of CD4^+^ cells in tumors of C57BL/6 mice. Scale bar, 100 μm. (I) Representative images of aSMA and Sirius Red staining in tumors from C57BL/6 mice (*n* = 5). Scale bar, 100 μm.

Supplementary Material 3: Figure S3. Decreased glycolysis can attenuate the migration and invasion of cancer cells. (A) qRT-PCR analysis of *Ldha* mRNA expression levels in shNC, shLDHA-1, shLDHA-2, and shLDHA-3 cells. The results are relative to the expression levels in shNC cells after normalization to *β-actin* mRNA expression (*n* = 3). (B) Representative FCM plots (left) and quantification of 2-NBDG staining (right) in shNC and shLDHA cells (*n* = 3). ‘Relative MFI’ denotes 2-NBDG MFI of stained samples relative to the matched unstained cells. (C, D) Representative images of shNC and shLDHA cells hematoxylin and eosin (H&E) staining, and the numbers of (C) migrating and (D) invading cells (*n* = 3, migration for 24 h and invasion for 48 h). (E–G) FCM analysis of the percentages of tumor-infiltrating (E) PMN-MDSCs, (F) TAMs, and (G) CD4^+^ T cells among the CD45^+^ cells of shNC and shLDHA tumors (*n* = 5) from C57BL/6 mice. (H) Representative IHC staining of CD4^+^ cells in tumors of C57BL/6 mice. Scale bar, 100 μm. (I) Representative images of aSMA and Sirius Red staining in tumors from C57BL/6 mice (*n* = 5). Scale bar, 100 μm.

Supplementary Material 4: Figure S4. Sc-RNA-seq can help classify CD45^+^ cells and DCs into clusters. A UMAP plots showing the locations of CD45^+^ cells in shNC and shLDHA tumors. B Violin plots showing the relative expression levels of marker genes with high differential expression among the eleven clusters in shNC and shLDHA tumors. C, D UMAP plots showing the expression levels of representative genes in (C) the eleven clusters of CD45^+^ cells and in (D) the four clusters of DCs. E The UMAP plots of 381 T cells were classified into two subsets using known marker genes. F The dot plots of representative genes related to T cell subsets and Z-scores normalized log2. The center represents the average expression level across all single cells with a color scale from 0.4 to -0.4. G, H The violin plots showing the (G) TCA signature score and (H) average Sl2ac3 expression in CD8^+^ T cells.

Supplementary Material 5. Figure S5. An overview of the co-culture system and the gating strategies. A Overview of the ALI-3D co-culture system. B Representative gating strategies for BM-DCs in ALI-3D. C–F Representative FCM plots of MHC I, MHC II, CD80 and CD86. C The FCM plots of BM-DCs ALI-3D co-cultured with control and IG cells (*n* = 3). D The FCM plots of BM-DCs ALI-3D co-cultured with shNC and shLDHA cells (*n* = 3). E The FCM plots of BM-DCs cultured with control and IG supernatants (*n* = 3). F The FCM plots of BM-DCs cultured with shNC and shLDHA supernatants (*n* = 3).

Supplementary Material 6: Figure S6. BM-DC differentiation process and FCM analysis. A An overview of methods for differentiation of C57BL/6 mice-derived bone marrow cells into BM-DCs and an overview of IF methods for BM-DCs. B Representative gating strategies for BM-DCs cultured in control and CM RPMI. C The FCM plots of BM-DCs cultured with control RPMI and CM RPMI without LPS (*n* = 3). D The FCM plots of BM-DCs cultured with control RPMI and CM RPMI with LPS (*n* = 3).

Supplementary Material 7: Table S1. The DCs differentially expressed genes identified by scRNA-seq in shNC and shLDHA tumors.

Supplementary Material 8: Table S2. Antigen presentation gene significance score, TCA signature score and Cytotoxic signature score for CD8 + T cell in scRNA-seq.

## Data Availability

The scRNA-seq data generated in this study are publicly available in Gene Expression Omnibus (GEO) at GSE254189.

## Abbreviations

PDAC: Pancreatic ductal adenocarcinoma
DC: Dendritic cell
TME: Tumor microenvironment
scRNA-seq: Single-cell RNA sequencing
BM-DC: Bone marrow-derived dendritic cell
cDC: Conventional dendritic cell
ICB: Immune checkpoint blockade
IFN-γ: Interferon gamma
DMEM: Dulbecco’s Modified Eagle Medium
FBS: Fetal bovine serum
IG: Increased glycolysis cancer cell
qRT-PCR: Quantitative reverse transcription polymerase chain reaction
RPMI: Roswell Park Memorial Institute
GM-CSF: Granulocyte/macrophage-colony stimulating factor
FLT3-L: Flt3-Ligand
FCM: Flow cytometry
ALI-3D: Air–Liquid Interface 3-Dimensional
PDO: PDAC organoid
H&E: Hematoxylin and eosin
IHC: Immunohistochemistry
IF: Immunofluorescence
OCR: Oxygen consumption rate
ECAR: Extracellular acidification rate
GO: Gene Ontology
GSEA: Gene Set Enrichment Analysis
OS: Overall survival
Mo-MDSC: Monocytic myeloid-derived suppressor cell
PMN-MDSC: Polymorphonuclear myeloid-derived suppressor cell
TAM: Tumor-associated macrophage
OXPHOS: Oxidative phosphorylation
TLR: Toll-like receptor
LPS: Lipopolysaccharide
