# Supplementary figures and images for: Dynamic glycolytic reprogramming effects on dendritic cells in pancreatic ductal adenocarcinoma
Source: J Exp Clin Cancer Res. 2024 Sep 30;43:271. doi: 10.1186/s13046-024-03192-8 (PMC11441259; doi:10.1186/s13046-024-03192-8)

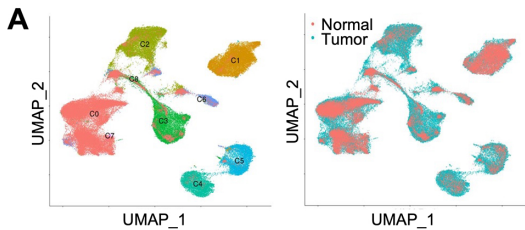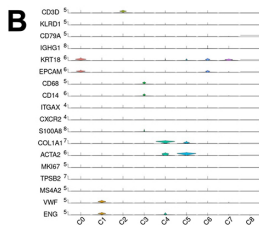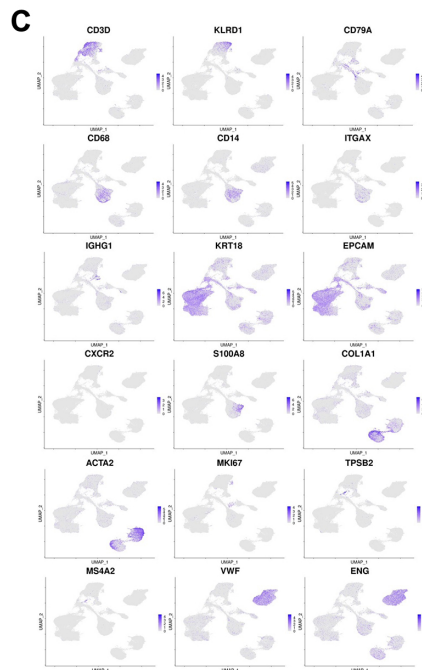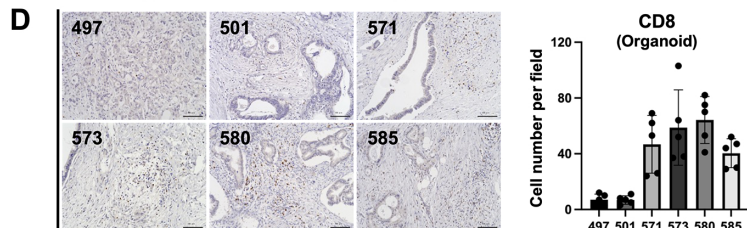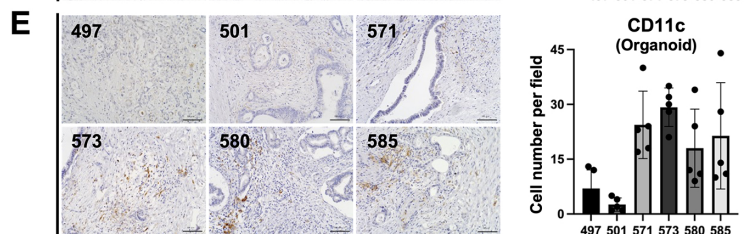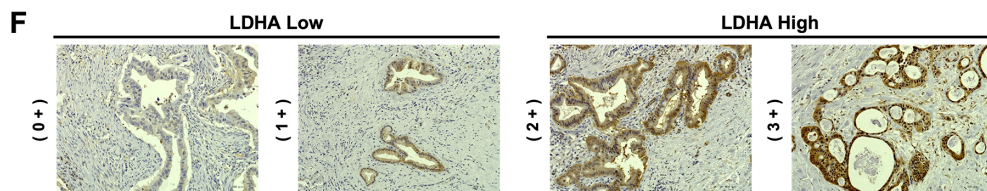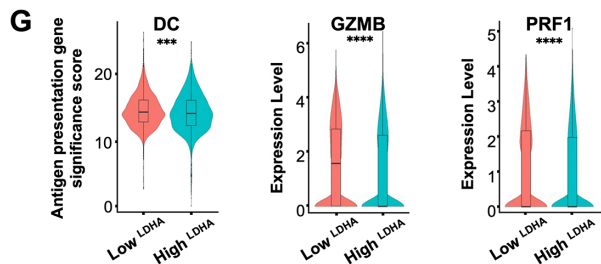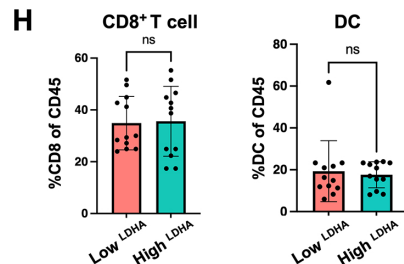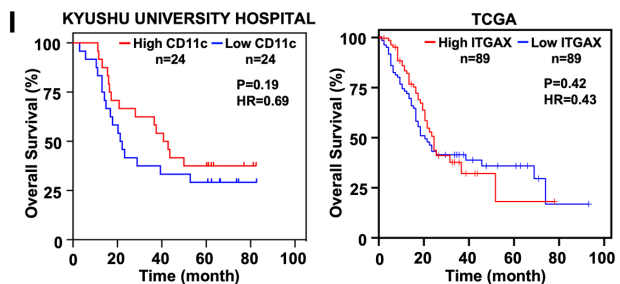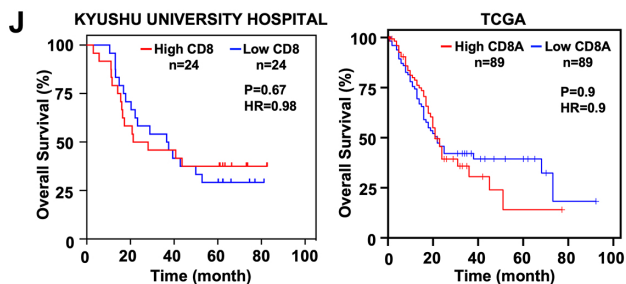

Supplement: Supplementary file 1 — Supplementary Material 1: Figure S1. Public scRNA-seq data analysis and IHC staining. (A) We analyzed public scRNA-seq data and found that the UMAP plots of 163,830 cells from primary pancreatic tumors of PDAC patients (Tumor) and normal pancreatic tissues of patients without PDAC (Normal) were classified into nine subsets using known marker genes. (B) Violin plots showing the relative expression levels of marker genes with high differential expression among the nine clusters. (C) UMAP plots showing the expression levels of representative genes in the nine clusters. (D, E) Representative IHC staining of (D) CD8+ cells and (E) CD11c+ cells corresponding to PDOs. Scale bar, 100 μm. (F) LDHA expression IHC scoring levels in PDAC samples: 0+ and 1+ scores are the low LDHA expression group and 2+ and 3+ scores are the high LDHA expression group. (G) Analysis of public scRNA-seq data revealed an increased antigen presentation gene significance score in DCs, as well as higher GZMB and PRF1 expression levels, in CD8+ T cells from primary pancreatic tumors of PDAC patients with low LDHA expression levels (Low LDHA) compared with those from PDAC tumors with high LDHA expression levels (High LDHA). (H) Analysis of public scRNA-seq data revealed no significant difference in the proportion of DCs or CD8+ T cells among the CD45+ cells between PDAC tumors with low LDHA expression levels (Low LDHA) and high LDHA expression levels (High LDHA). (I, J) OS analyses (Kaplan–Meier curve analysis) of PDAC patients were performed based on the expression levels of (I) CD11c (ITGAX) and (J) CD8 (CD8A), using both our cohort data and TCGA datasets. [file 13046_2024_3192_MOESM1_ESM.pdf]

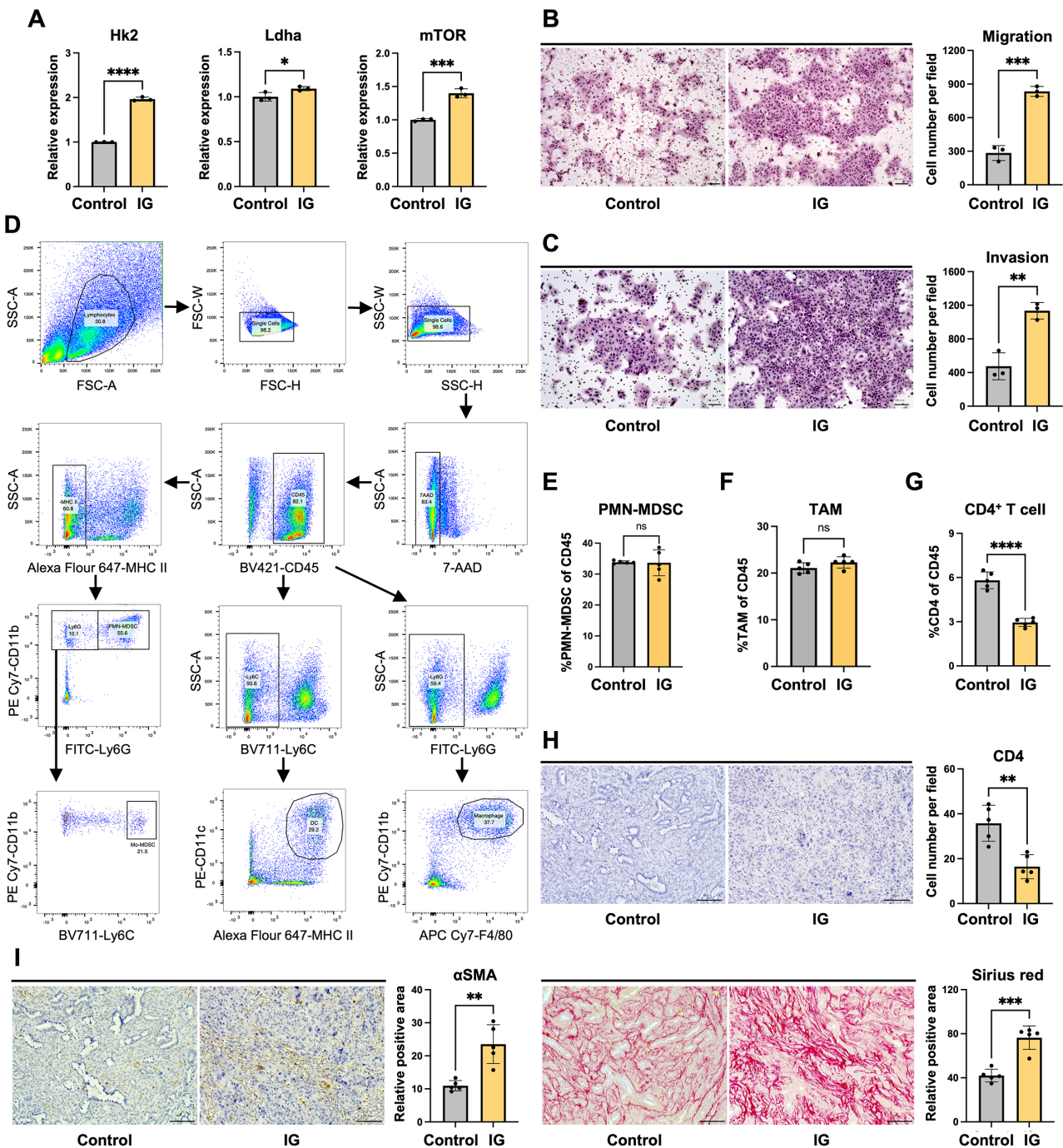

Supplement: Supplementary file 2 — Supplementary Material 2: Figure S2. Increased glycolysis can enhance the migration and invasion of cancer cells. (A) qRT-PCR analysis of the Hk2, Ldha and mTOR mRNA expression levels in control and increased glycolysis (IG) cells. The expression levels are relative to those in the control cells and were normalized to β-actin mRNA expression (n = 3). (B, C) Representative images of control and IG cell hematoxylin and eosin (H&E) staining, and the number of (B) migrating and (C) invading cells (n = 3, migration for 24 h and invasion for 48 h). (D) Representative gating strategies for tumor-infiltrating DCs, PMN-MDSCs, Mo-MDSCs, and TAMs. (E–G) FCM analysis of the percentages of tumor-infiltrating (E) PMN-MDSCs, (F) TAMs, and (G) CD4+ T cells among the CD45+ cells of control and IG tumors (n = 5) from C57BL/6 mice. (H) Representative IHC staining of CD4+ cells in tumors of C57BL/6 mice. Scale bar, 100 μm. (I) Representative images of aSMA and Sirius Red staining in tumors from C57BL/6 mice (n = 5). Scale bar, 100 μm. [file 13046_2024_3192_MOESM2_ESM.pdf]

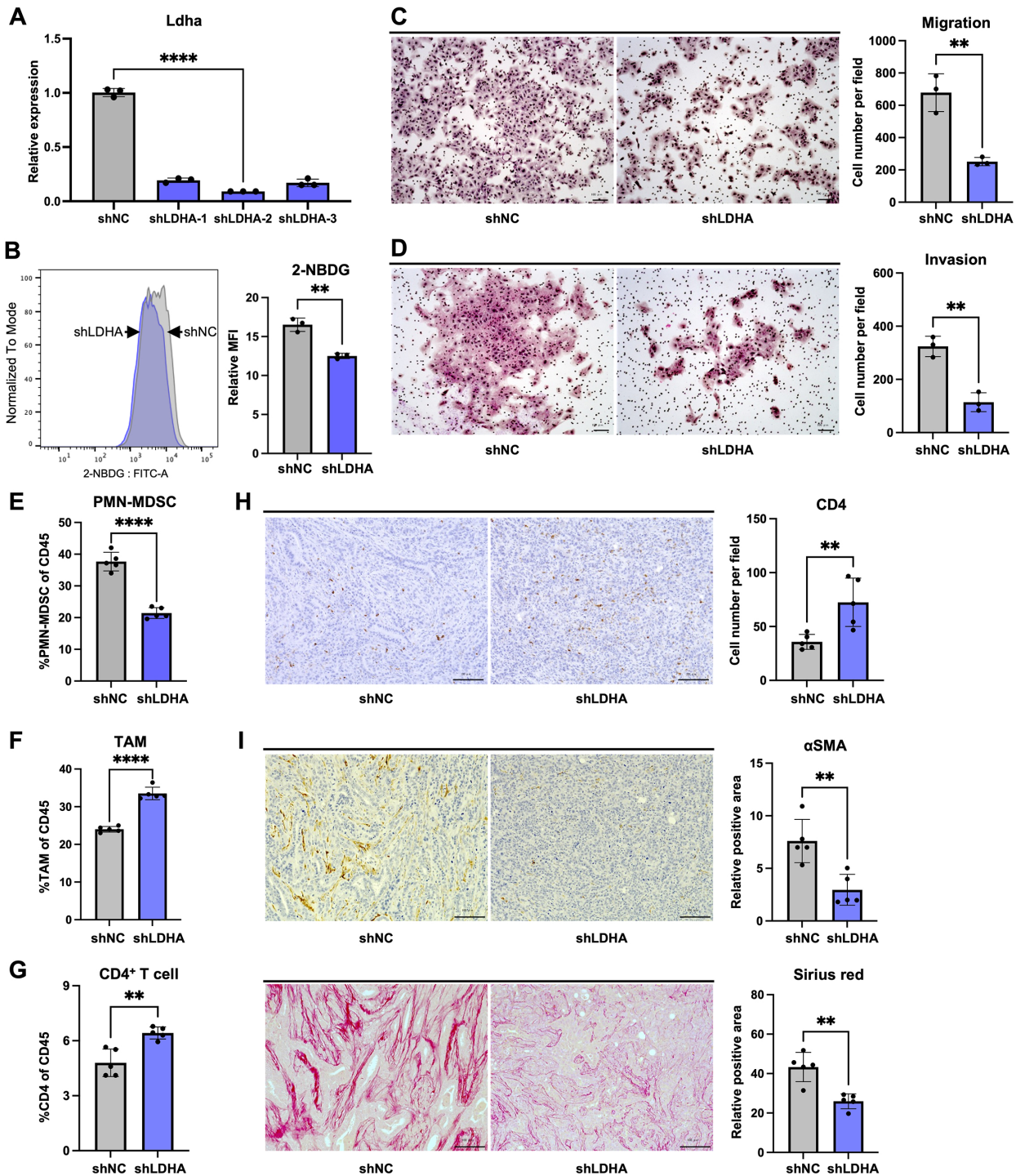

Supplement: Supplementary file 3 — Supplementary Material 3: Figure S3. Decreased glycolysis can attenuate the migration and invasion of cancer cells. (A) qRT-PCR analysis of Ldha mRNA expression levels in shNC, shLDHA-1, shLDHA-2, and shLDHA-3 cells. The results are relative to the expression levels in shNC cells after normalization to β-actin mRNA expression (n = 3). (B) Representative FCM plots (left) and quantification of 2-NBDG staining (right) in shNC and shLDHA cells (n = 3). ‘Relative MFI’ denotes 2-NBDG MFI of stained samples relative to the matched unstained cells. (C, D) Representative images of shNC and shLDHA cells hematoxylin and eosin (H&E) staining, and the numbers of (C) migrating and (D) invading cells (n = 3, migration for 24 h and invasion for 48 h). (E–G) FCM analysis of the percentages of tumor-infiltrating (E) PMN-MDSCs, (F) TAMs, and (G) CD4+ T cells among the CD45+ cells of shNC and shLDHA tumors (n = 5) from C57BL/6 mice. (H) Representative IHC staining of CD4+ cells in tumors of C57BL/6 mice. Scale bar, 100 μm. (I) Representative images of aSMA and Sirius Red staining in tumors from C57BL/6 mice (n = 5). Scale bar, 100 μm. [file 13046_2024_3192_MOESM3_ESM.pdf]

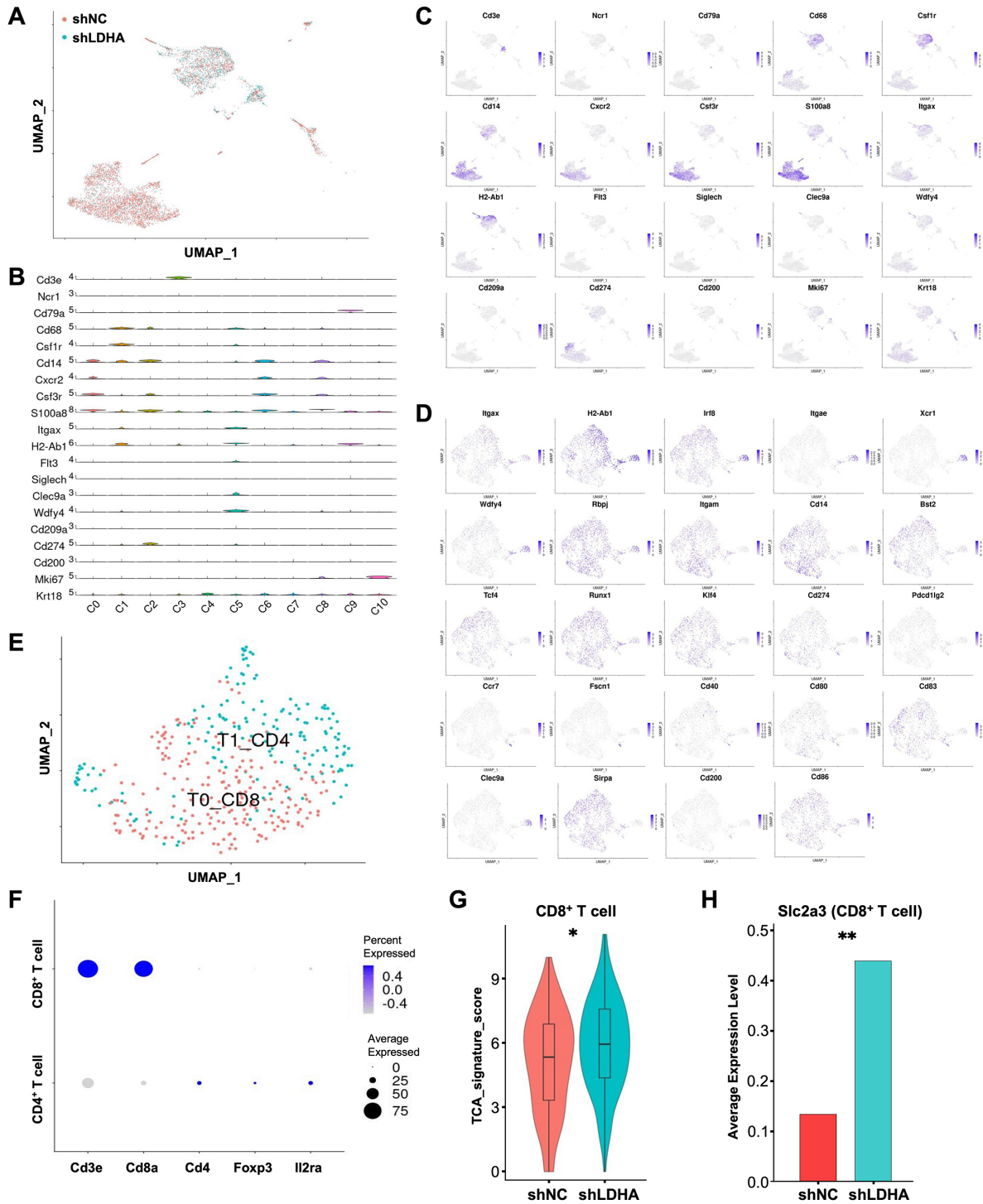

Supplement: Supplementary file 4 — Supplementary Material 4: Figure S4. Sc-RNA-seq can help classify CD45+ cells and DCs into clusters. A UMAP plots showing the locations of CD45+ cells in shNC and shLDHA tumors. B Violin plots showing the relative expression levels of marker genes with high differential expression among the eleven clusters in shNC and shLDHA tumors. C, D UMAP plots showing the expression levels of representative genes in (C) the eleven clusters of CD45+ cells and in (D) the four clusters of DCs. E The UMAP plots of 381 T cells were classified into two subsets using known marker genes. F The dot plots of representative genes related to T cell subsets and Z-scores normalized log2. The center represents the average expression level across all single cells with a color scale from 0.4 to -0.4. G, H The violin plots showing the (G) TCA signature score and (H) average Sl2ac3 expression in CD8+ T cells. [file 13046_2024_3192_MOESM4_ESM.pdf]

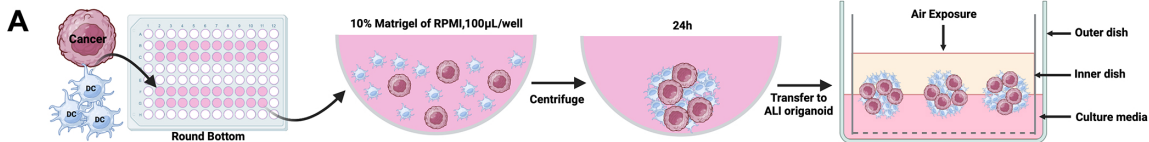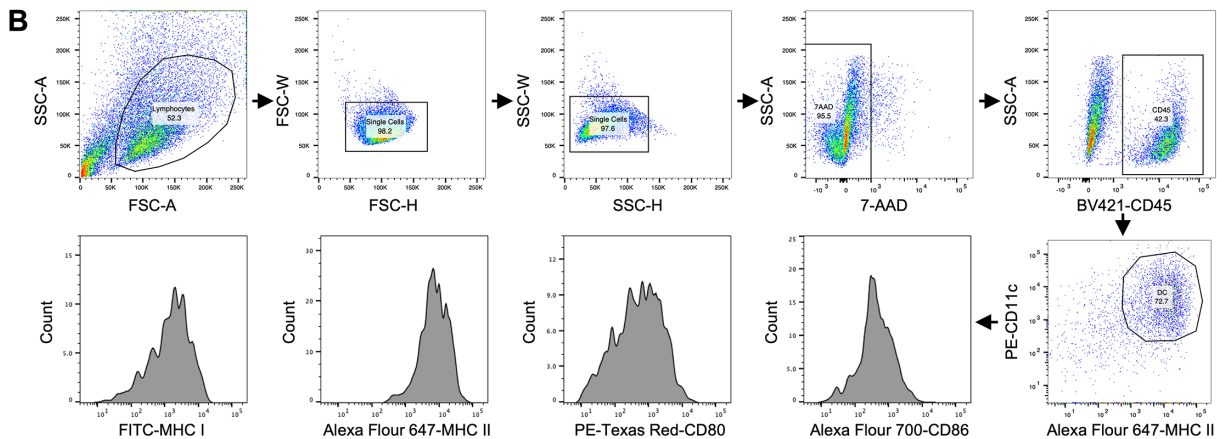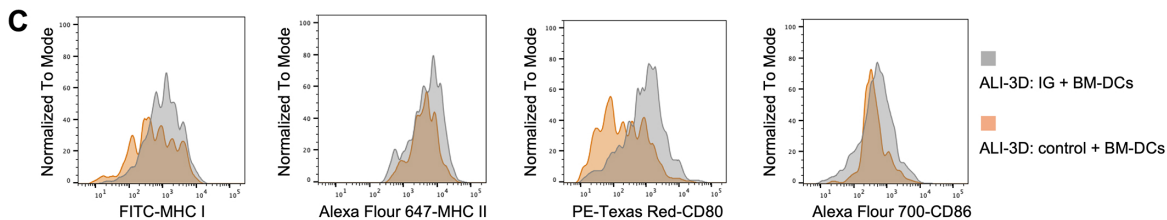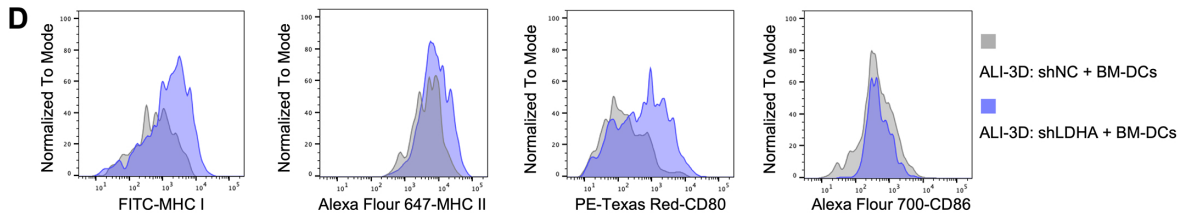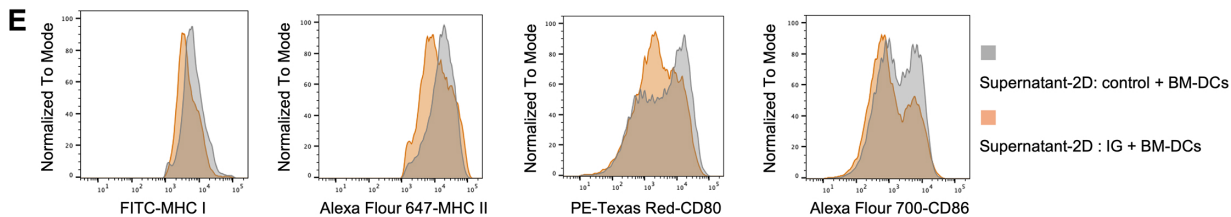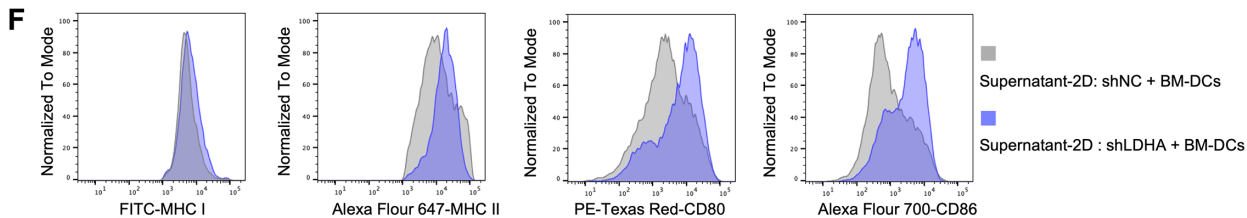

Supplement: Supplementary file 5 — Supplementary Material 5. Figure S5. An overview of the co-culture system and the gating strategies. A Overview of the ALI-3D co-culture system. B Representative gating strategies for BM-DCs in ALI-3D. C–F Representative FCM plots of MHC I, MHC II, CD80 and CD86. C The FCM plots of BM-DCs ALI-3D co-cultured with control and IG cells (n = 3). D The FCM plots of BM-DCs ALI-3D co-cultured with shNC and shLDHA cells (n = 3). E The FCM plots of BM-DCs cultured with control and IG supernatants (n = 3). F The FCM plots of BM-DCs cultured with shNC and shLDHA supernatants (n = 3). [file 13046_2024_3192_MOESM5_ESM.pdf]

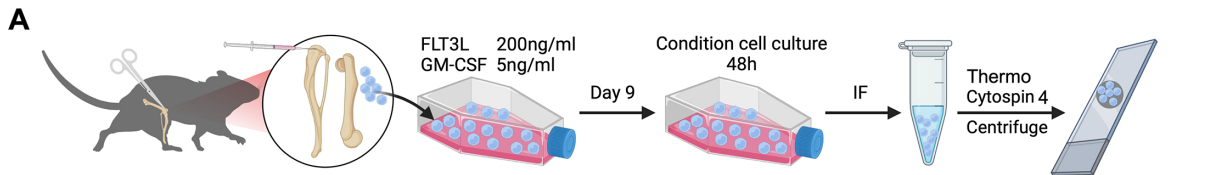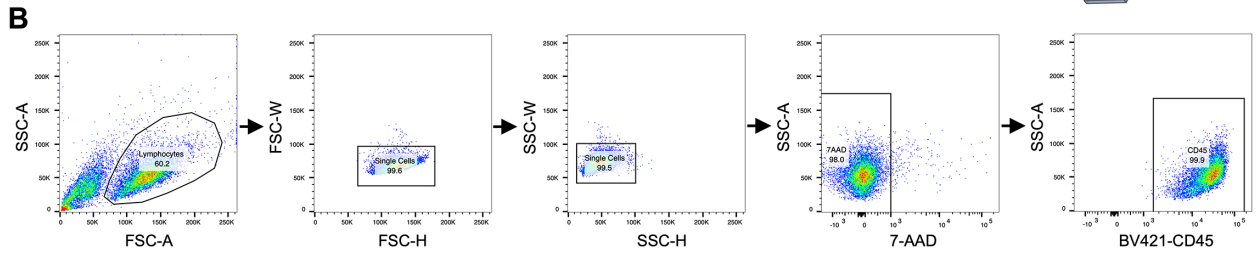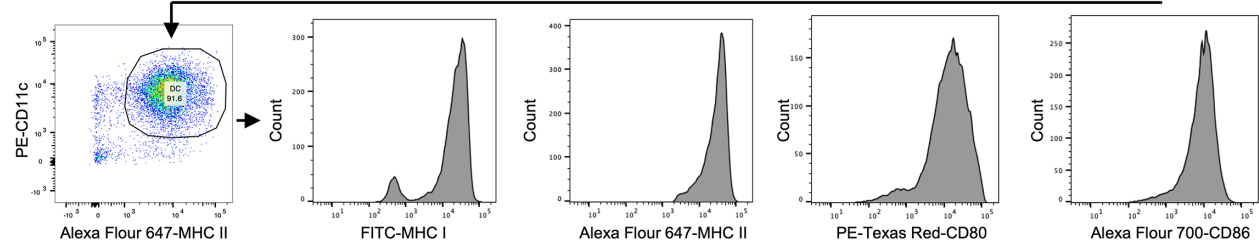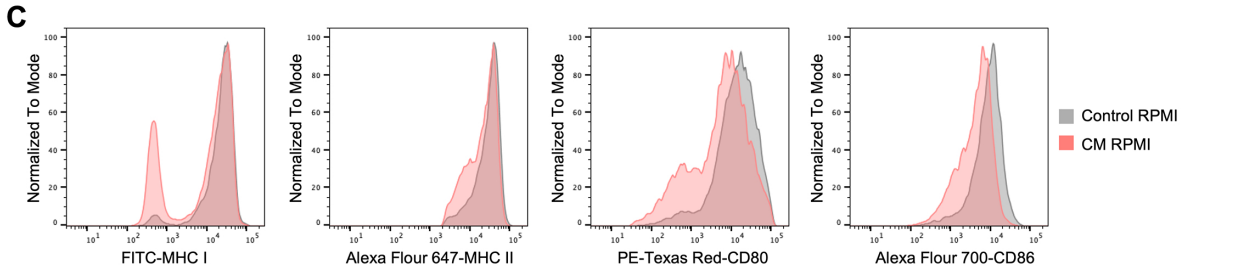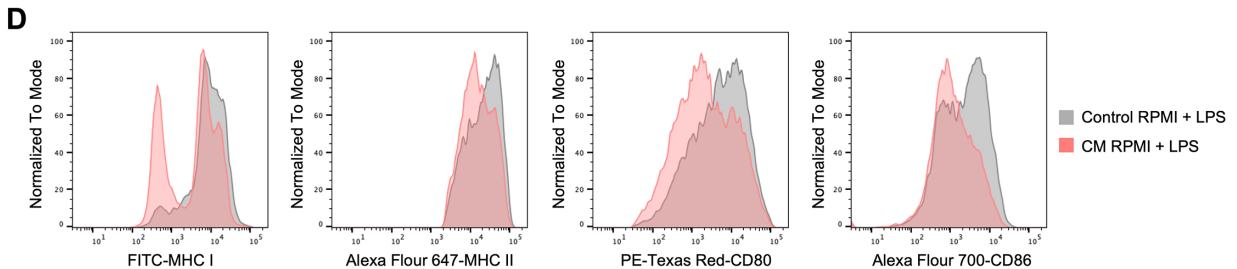

Supplement: Supplementary file 6 — Supplementary Material 6: Figure S6. BM-DC differentiation process and FCM analysis. A An overview of methods for differentiation of C57BL/6 mice-derived bone marrow cells into BM-DCs and an overview of IF methods for BM-DCs. B Representative gating strategies for BM-DCs cultured in control and CM RPMI. C The FCM plots of BM-DCs cultured with control RPMI and CM RPMI without LPS (n = 3). D The FCM plots of BM-DCs cultured with control RPMI and CM RPMI with LPS (n = 3). [file 13046_2024_3192_MOESM6_ESM.pdf]
